# piRNAs Are Associated with Diverse Transgenerational Effects on Gene and Transposon Expression in a Hybrid Dysgenic Syndrome of *D*. *virilis*

**DOI:** 10.1371/journal.pgen.1005332

**Published:** 2015-08-04

**Authors:** Alexandra A. Erwin, Mauricio A. Galdos, Michelle L. Wickersheim, Chris C. Harrison, Kendra D. Marr, Jack M. Colicchio, Justin P. Blumenstiel

**Affiliations:** 1 Department of Ecology and Evolutionary Biology, University of Kansas, Lawrence, Kansas, United States of America; 2 Department of Molecular Biosciences, University of Kansas, Lawrence, Kansas, United States of America; Fred Hutchinson Cancer Research Center, UNITED STATES

## Abstract

Sexual reproduction allows transposable elements (TEs) to proliferate, leading to rapid divergence between populations and species. A significant outcome of divergence in the TE landscape is evident in hybrid dysgenic syndromes, a strong form of genomic incompatibility that can arise when (TE) family abundance differs between two parents. When TEs inherited from the father are absent in the mother's genome, TEs can become activated in the progeny, causing germline damage and sterility. Studies in *Drosophila* indicate that dysgenesis can occur when TEs inherited paternally are not matched with a pool of corresponding TE silencing PIWI-interacting RNAs (piRNAs) provisioned by the female germline. Using the *D*. *virilis* syndrome of hybrid dysgenesis as a model, we characterize the effects that divergence in TE profile between parents has on offspring. Overall, we show that divergence in the TE landscape is associated with persisting differences in germline TE expression when comparing genetically identical females of reciprocal crosses and these differences are transmitted to the next generation. Moreover, chronic and persisting TE expression coincides with increased levels of genic piRNAs associated with reduced gene expression. Combined with these effects, we further demonstrate that gene expression is idiosyncratically influenced by differences in the genic piRNA profile of the parents that arise though polymorphic TE insertions. Overall, these results support a model in which early germline events in dysgenesis establish a chronic, stable state of both TE and gene expression in the germline that is maintained through adulthood and transmitted to the next generation. This work demonstrates that divergence in the TE profile is associated with diverse piRNA-mediated transgenerational effects on gene expression within populations.

## Author Summary

Transposable elements (TEs) are selfish elements that copy themselves. More than half of the human genome is comprised of such elements. Studies in the fruit flies *Drosophila melanogaster* and *D*. *virilis* have been important in demonstrating a role for RNA silencing by PIWI-interacting RNAs (piRNAs) in protecting the genome against these harmful elements. These small RNAs are capable of recognizing TE mRNAs and mediating their destruction. They are also transmitted by the female germline to offspring in order to maintain a stable genome across generations. When males carrying a particular TE family are crossed with females lacking the element, the mother is unable to provide genome defense via complementary piRNAs that target the element. This leads to excess TE activation in the germline and sterility, a phenomenon known as hybrid dysgenesis. In this article we characterize the genomic landscape of TE destabilization that occurs in dysgenic crosses of *D*. *virilis*. We demonstrate that this mobilization is associated with an increased level of germline TE expression that persists through adulthood. In addition, we find that TE activation is associated with diverse effects on normal gene expression that are also mediated by piRNAs.

## Introduction

In sexually reproducing species, two unique haploid genomes join together in syngamy to establish each generation. This mixing of genomes introduces potentially advantageous variation under changing environmental conditions, but also provides a condition ripe for exploitation by selfish elements [1]. Because syngamy can introduce selfish elements to new genomes and recombination can separate selfish elements from their harmful consequences, selfish elements such as transposable elements (TEs) can proliferate [2,3]. This is exemplified by the *P* element in *Drosophila melanogaster*. Through a likely horizontal transfer event from the distant species *D*. *willistoni*, the *P* element invaded *D*. *melanogaster* less than 100 years ago and is now found in *D*. *melanogaster* world-wide [4,5].

Because TEs can be harmful and also drive a rapid accumulation of differences between species, they have been proposed to contribute to reproductive isolation. While their proliferative nature makes it very unlikely that TEs are drivers of speciation itself [6,7], TE misregulation has been observed in a variety of interspecific hybrids. For example, increased TE expression is observed in malformed backcrosses between recently diverged species of lake whitefish [8]. Similar observations have been made in species ranging from *Arabidopsis* [9] to wallaby [10,11]. Studies in *Drosophila* using interspecific crosses have been especially important for our understanding of TE control in hybrids. The results so far have been idiosyncratic. Interspecific hybrids between closely related members of the *affinis*, *simulans*, *virilis* and *pseudoobscura* groups of *Drosophila* show little evidence for increased transposition [6,7,12]. In contrast, increased transposition is observed in crosses between *D*. *buzzatii* and *D*. *koepferae* [13,14]. In the latter case, the increased rate of TE movement has been attributed to a form of genomic stress, though the nature of this stress is not clear. Additionally, interspecific hybrids between *D*. *simulans* and *D*. *melanogaster* (which are more distantly related compared to those in previous crosses examining this question [6,7]) do show increased expression of TEs [15] and this is attributed to adaptive divergence in components of the TE regulatory machinery. Since species may differ significantly both in TE profile and regulatory machinery protein function, it is challenging to determine how divergence in TE profile alone contributes to TE activation in interspecific hybrids.

For this reason, intraspecific syndromes of hybrid dysgenesis provide critical insight into the role that divergence in TE profile can play in determining TE activity across generations. Hybrid dysgenesis is defined as a syndrome of hybrid sterility [16,17] and germline damage that occurs in intraspecific crosses when the male carries one or more TE families absent in the female [18–20]. The dysgenesis phenomenon in *Drosophila* has provided crucial insight into mechanisms of host genome defense by small RNAs. This is because activation of TEs inherited solely through the *Drosophila* male germline can be explained by the fact that the maternal germline is the primary agent of transgenerational TE repression via PIWI-interacting RNAs (piRNAs) maternally loaded into the egg [21].

piRNAs are 23–30 nt RNAs found in complex with PIWI proteins and they play a crucial role in maintaining genome integrity via the repression of TEs. Many piRNAs are derived from TE fragments residing in distinct genomic regions known as piRNA clusters [22–24]. Anti-sense TE transcripts derived from these clusters are processed into piRNAs and, in complex with PIWI proteins, serve as guides to target resident TE transcripts for PIWI-mediated 'slicing' [25]. This system serves as a mechanism of genome defense because the proliferative nature of TEs can be inherently recognized by their tendency to transpose into piRNA clusters, whereby they serve as guides to recognize mRNAs of the same TE family. In the absence of the maternally provisioned piRNAs that target TE mRNAs for PIWI-mediated slicing, paternally inherited TEs become activated in the progeny germline. This has been demonstrated for the *P-M* and *I-R* systems of hybrid dysgenesis in *D*. *melanogaster* [24,26–28]. In the *P-M* system, *P* strains, but not *M* strains, carry active copies of the DNA transposon known as the *P* element. In the *I-R* system, *I* (*Inducer*) strains, but not *R* (*Reactive*) strains, carry active copies of the non-LTR retrotransposon *I* element. Dysgenesis arises when *I* or *P* strain males are mated, respectively, with *R* or *M* strain females lacking such elements. These females are unable to maternally provision matching piRNAs that target the activating element.

The *P-M* and *I-R* systems of dysgenesis represent cases in which the TE profile differs between strains with respect to only one family of inducing elements. In contrast, a third syndrome of hybrid dysgenesis in *D*. *virilis* represents a more complicated form of dysgenesis that appears to be caused by the mass action of multiple active elements abundant in one strain, but not another. Elements that likely contribute to this syndrome were first identified through direct analysis of induced lesions identified in the offspring of F1 progeny that escaped sterility from the dysgenic cross. The major driver of dysgenesis appears to be the *Penelope* element, the founding member of a clade of retroelements designated *Penelope-*like elements that are distinct from non-LTR and LTR retroelements [29]. Active copies of *Penelope* are abundant in the inducer strain (Strain 160) and only degenerate copies are present in the *M*/*R-*like reactive strain (Strain 9) [30]. Furthermore, expression of the *Penelope* element is elevated in the ovaries and testes of F1 dysgenic progeny that have escaped ablation of the gonads [31]. In addition to the *Penelope* element, three other elements (*Helena*, a non-LTR*; Paris* and *Polyphemus*, both DNA transposons [32,33]) are also more abundant in the 160 inducer strain, and these likely contribute to dysgenesis. A complex mode of hybrid dysgenesis, driven jointly by multiple elements, is supported by the fact that females of some "neutral" strains—capable of preventing dysgenesis when crossed with inducer males but also incapable of induction [34]–lack *Penelope* piRNAs in the their germline. If *Penelope* is the sole cause of paternal induction, it is difficult to explain how such strains could prevent induction when the female germline lacks *Penelope* piRNAs.

In light of this complexity, a second model for hybrid dysgenesis not directly driven by transposable elements in *D*. *virilis* has been proposed. A previous study showed that not only do inducer and reactive strains differ with respect to TE abundance, they also differ with respect to piRNA cluster activity [35]. In particular, small RNA sequencing in these two strains demonstrated that telomeric regions of the inducer strain exhibit uniquely strong piRNA cluster activity. Differences in telomeric cluster activity were proposed as potentially causative of dysgenesis. Here, we directly test this hypothesis by genetically assessing the contribution of inducer strain telomeres to hybrid sterility.

Because the 160 inducer strain and the 9 reactive strain of *D*. *virilis* differ with respect to multiple elements, the dysgenic syndrome in *D*. *virilis* may perhaps be more similar to that observed between species with respect to TE profiles, but with minimal divergence in protein coding function since it arises from an intraspecific cross. This syndrome may be considered an intermediate state between the *P-M* and *I-R* models and crosses between entirely different species that differ significantly at the genic level as well. Therefore, the dysgenic syndrome in *D*. *virilis* serves as a useful model for understanding the consequences of accumulating differences in TE profile between populations.

A fundamental question is how TE activation in dysgenic crosses influences the entire genomic TE landscape. Early studies of *P* element hybrid dysgenesis in *D*. *melanogaster* indicated downstream activation of additional TEs [36], but this interpretation was soon called into doubt [37]. Nonetheless, the syndrome of hybrid dysgenesis in *D*. *virilis* provides strong support for cascading germline activation of TEs because multiple TEs were found to transpose in the germline of dysgenic progeny [31,38]. While different TE families may contribute to the initial induction of dysgenesis in *D*. *virilis*, germline co-mobilization has been demonstrated by the transposition of TEs that are evenly distributed between the two strains, in contrast to *Penelope*, *Helena* and *Paris*, which are more abundant in inducer strain 160 [32,39,40]. Significantly, a recent study of the *P* element system indicates that the previous conclusion of no co-mobilization may have been premature [28]. In the face of *P* element activation, DNA damage can perturb piRNA biogenesis and this defective piRNA biogenesis is presumed to drive the mobilization of additional TEs. Thus, global TE mobilization may also be observed in syndromes of hybrid dysgenesis that are driven by a single element. Whether a similar mechanism caused by DNA damage explains co-mobilization in the *D*. *virilis* system is unknown.

To fully understand the mechanisms underlying TE activation in dysgenic crosses, the developmental context must be considered since co-mobilization may be induced at any point in the developing or aging germline. A critical feature of the *P-M* system is that the germline crisis ameliorates with age. In particular, as flies age to 21 days, fertility is partially restored and piRNA levels that target the *P* element are restored to the same level as non-dysgenic reciprocal females. Thus, even though *P* element derived piRNAs are not maternally inherited, *de novo* piRNA production from paternally inherited P insertions is evident. This *de novo* piRNA production also coincides with restored silencing of *P* element mRNA. Rescue of germline crisis is also proposed to be enhanced by movement of other elements into piRNA clusters [28].

Here we use the unique system of hybrid dysgenesis in *D*. *virilis* to define the landscape of TE expression that coincides with the initial activation of multiple TE families. Because analysis of piRNA production and TE expression can be confounded in atrophied gonads, we focus solely on germline tissues that have escaped complete gonadal atrophy. Specifically, we examine piRNA and TE expression in 0–2 hour old embryos laid by F1 females from non-dysgenic crosses, and F1 escaper females of the dysgenic cross. This represents an endpoint of the dysgenic crisis and also provides insight into how the effects of hybrid dysgenesis in females that escape sterility can be passed on to further generations. In contrast to the *P-M* system, which may resolve within the germline as flies age [28], the effects of dysgenesis on TE expression in the *D*. *virilis* system persist through adulthood. Because this occurs within an intraspecific cross, increased levels of persisting TE expression are not explained by divergence in the piRNA machinery.

By comparing 0–2 hour old embryos laid by genetically identical females derived from dysgenic versus non-dysgenic crosses, we show that germline activation of TEs is driven by a multi-layered mechanism. Diverse elements are activated corresponding to TE copy number asymmetry between strains but there is also corresponding activation of some TEs that are evenly distributed between strains. This state of chronic increased TE expression is maintained as flies age, suggesting a different mechanism underlying co-mobilization compared to the *P-M* system. Interestingly, increased and persistent TE expression in the germline of females of the dysgenic cross coincides with a shift in piRNA pools. This shift in piRNA pools is associated with increased abundance of piRNAs that target genes outside of piRNA clusters, leading to significant effects on non-TE gene expression. Finally, differences in the TE profile between strains coincide with different modes of trans-generational gene regulation by genic piRNAs that arise from polymorphic TE insertions. Overall, this work identifies multiple modes by which differences in the TE landscape between strains can influence patterns of TE and gene expression across generations via piRNAs.

## Results

### Genome wide asymmetry in TE abundance in a dysgenic cross of *D*. *virilis*

**Fig 1:**
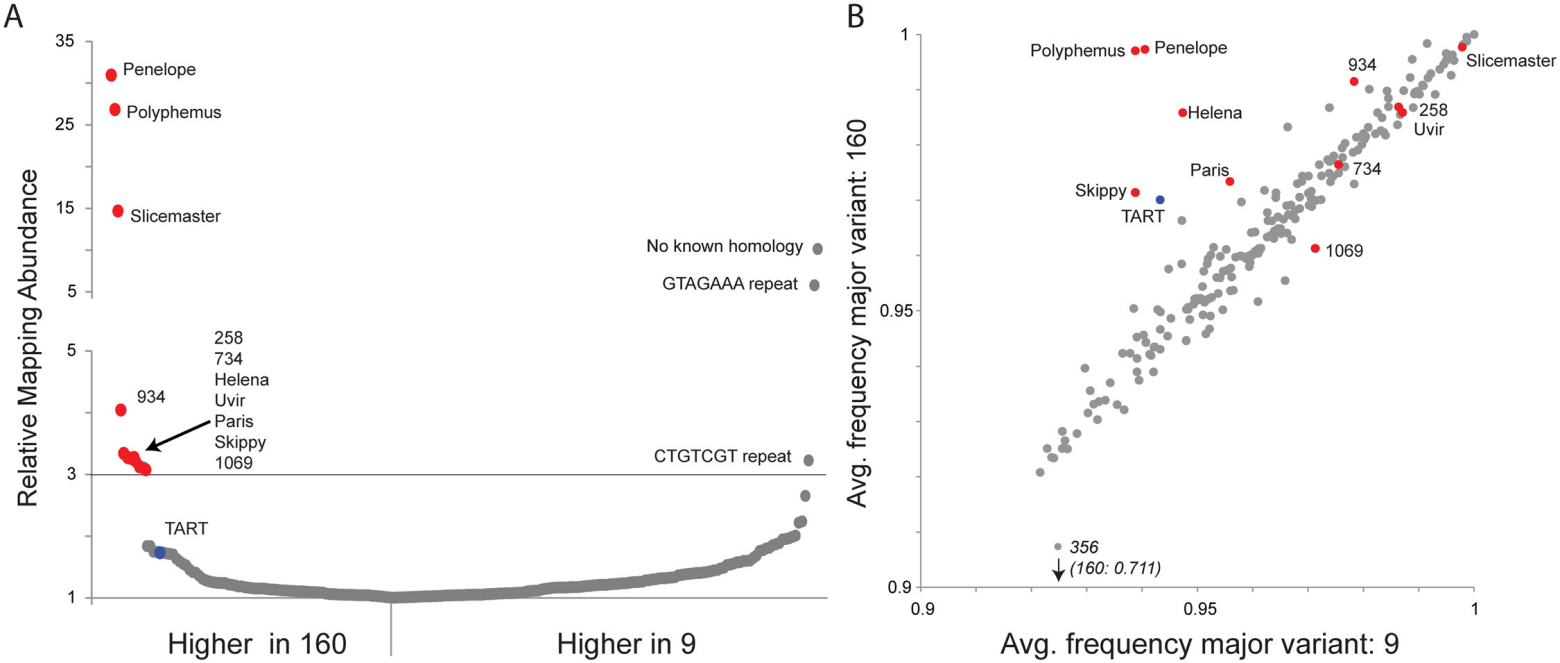
Multiple transposable elements are associated with induction of hybrid dysgenesis. (A) Relative mapping abundance of single-end, 100 bp reads from strain 9 and strain 160 (normalized by reads mapping to the genome), to a consolidated repeat library. Eleven elements are in 3-fold excess in strain 160 and are indicated here and throughout with red. TART elements are about 1.7-fold in excess and are indicated here and throughout with blue. No apparent TEs were found in excess in strain 9. (B) Using piledriver (https://github.com/arq5x/piledriver) we assessed homogeneity within reads mapping to the TE library by determining the average frequency of the major variant in both strains. TEs in excess in strain 160 are either more homogenous in strain 160 or similarly aged between strains, with the exception of element 1069 which shows slightly more homogeneity in strain 9.

Previous studies identified *Penelope* to be the primary driver of dysgenesis in the *D*. *virilis* system because multiple active copies reside in the inducer strain 160, but only degenerate copies reside in the reactive strain 9. *in situ* hybridization has identified more than 45 euchromatic *Penelope* insertions in strain 160 and none in strain 9 [32]. In addition, the *Helena* elements (euchromatic insertions Strain 160: 18; Strain 9: 0) and *Paris* elements (euchromatic insertions Strain 160: 26; Strain 9: 0) were shown to be more abundant in inducer strain 160 [32]. Recently a third element, *Polyphemus*, was identified as more abundant in strain 160. Using genome sequence reads from strains 160 and 9, mapped to a *D*. *virilis* TE/repeat library (S1 Dataset), we identified additional factors more abundant in strain 160 and potentially contributing to dysgenesis. Consistent with previous results, *Penelope* and *Polyphemus* showed the largest excess in strain 160, validating this approach. Using a 3-fold cutoff as a threshold, we further validated our detection method by confirming that *Helena* and *Paris* copy numbers are enriched in strain 160 [32]. Overall, we identified eleven elements enriched in strain 160 and three elements enriched in strain 9. Of the eleven enriched in strain 160, two repeat sequences (258 and 1069; Fig 1A) show no apparent evidence of TE related coding capacity (S2 Dataset). Likewise, the three repeat sequences enriched in strain 9 show no apparent evidence of TE related coding capacity. In addition to the four TEs known to be overrepresented in copy number in strain 160, and the two putatively non-TE repeat sequences, we identify five additional elements enriched in strain 160 (Fig 1A). These are candidates for contributing to the dysgenic syndrome. It is important to note that in this comparison there is a form of ascertainment bias. Because strain 160 is more closely related to the reference strain, repeats entirely absent from the reference strain (but possibly present in strain 9) will be excluded.

Coincident with the excess of multiple elements in strain 160, we found that the telomeric TART elements exhibit higher mapping abundance in strain 160, albeit below the 3-fold enrichment threshold (Fig 1A). TART elements have functioned as telomeres for millions of years in *Drosophila* [41], and our result demonstrates a strain-specific increase in bulk abundance of this long term resident. Telomeric TE content is under piRNA control [42–44], and previous work has shown increased piRNA cluster activity in the telomeric regions of strain 160 compared to strain 9 [35]. Since reduced piRNA function can lead to increased telomeric TE activity, our observed TART excess in strain 160 may be a readout of compromised piRNA function in the inducer strain that is either a cause or consequence of TE excess. For these reasons, it was critical to test for a role of telomeric regions in the induction of dysgenesis. Overall, we found that diverse TE families are in excess in strain 160. This finding is consistent with the hypothesis that the invasion of the *Penelope* element itself into the reactive strain contributes to genome instability, possibly through the co-mobilization of other TEs within the strain [45].

### TE age analysis identifies different modes for TE asymmetry between strains

Divergence in TE abundance between strains can result from different processes. For example, long-resident TEs may be in excess in one strain due to strain- or population-specific recent re-activation. By contrast, entirely new TEs may have invaded a species and have yet to spread equally throughout the genomes of the individuals within the population. The *P* element invasion in *D*. *melanogaster* is an example of the latter process [46]. It has recently invaded and is only present in natural populations/strains collected within the last seven decades.

To distinguish among alternative processes contributing to asymmetry in TE abundance between strains 160 and 9, we performed an age analysis of TE families using high sequence homogeneity within a TE family as an indicator of recent activation or invasion. A phylogenetic approach using full-length fragments is ideal for this purpose, but full-length TE assemblies are not available with short read sequencing technology. Therefore, we estimated relative TE family age by examining the sequence heterogeneity within mapping reads (Fig 1B) by considering the average frequency of the most common nucleotide variant, across all nucleotide variants within the mapping.

A young element that has recently invaded will show high similarity (higher homogeneity) among copies, nearing 1 for an average frequency of the major nucleotide variant. Older elements, with patterns of activation that occurred in the more distant past, accumulate sequence-level differences among insertions, which contribute to lower homogeneity. This accumulation of differences among multiple copies is evident by lower nucleotide frequencies of the most common variant. For a recent TE *re-activation* in only one strain, we expect higher sequence homogeneity within that strain but higher heterogeneity in the other strain, arising from degraded copies. For an element that has recently invaded a species and is present in both strains, but achieves greater copy number in one strain, we expect a similar level of sequence homogeneity in both strains. Finally, for an element that has recently invaded a species, but is entirely absent in one strain (similar to the *P* element in *D*. *melanogaster*), we expect higher homogeneity of reads in the carrying strain, but much higher heterogeneity in the naive strain, arising from sequence heterogeneity within the marginally mapping reads.

Our age analysis of TE families revealed two classes of TEs that are enriched in inducer strain 160 (Fig 1B). Consistent with previous analyses [30,33,47], one class includes *Penelope*, *Polyphemus*, *Helena* and *Paris*. In this class, we now also include *Skippy* and telomeric TART elements. *Penelope* and *Polyphemus* showed much higher homogeneity among copies in strain 160 compared to strain 9. We found the same pattern, albeit to a lesser extent, in *Helena*, *Paris*, *Skippy* and the telomeric TART elements. This pattern is highly consistent with recent activation of these elements from long-term resident status. A second class of elements exhibited a different pattern and included *Slicemaster*, *Uvir*, *258*, *734* and *1069*. *Slicemaster*, *Uvir*, *258* and *734* showed a pattern of nucleotide homogeneity consistent with similar age in both strains, while element *1069*, appears to be slightly older in strain 160. In the case of elements like *Slicemaster*, which are very young (>99% nucleotide similarity), this can be explained by recent invasion of both strains but excess movement in strain 160, rather than re-activation in one strain from long-time resident status.

### Increased germline TE expression in dysgenic females persists through adulthood

To determine the relationship between TE excess in strain 160 and TE expression in dysgenic progeny, we performed mRNA-seq from pooled 0–2 hour old embryos laid by F1 females of both the dysgenic (9 females X 160 males) and non-dysgenic (160 females X 9 males) directions of the cross. Notably, we did not measure TE expression in ovaries from F1 females because dysgenic ovaries are atrophied and expression analysis from these tissues is confounded by altered ratios of somatic and germline tissue. 0–2 hour old embryos laid by F1 mothers represent a sample of pure germline tissue, albeit lacking piRNAs and mRNAs residing solely in nurse cells that are not loaded into the egg [48]. This is because zygotic transcription in *D*. *virilis*, as measured with the early, zygotic *fushi-tarazu* (*ftz*) gene, begins after 2 hours [49]. Confirmation that embryos in these samples were collected prior to the onset of zygotic transcription was obtained by examining *ftz* expression in our RNA-seq dataset.

Full penetrance of dysgenesis, evidenced by fully atrophied gonads, is observed in approximately 50% of male and female progeny from 9 female X 160 male crosses. Therefore, embryos analyzed by mRNA-seq from the dysgenic cross were those laid by mothers that escaped full sterility. In contrast to other systems, hatch rates are normal in eggs laid by escaper females. For clarity, these tissues will be referred to as dysgenic, even though these tissues escaped complete atrophy. Sexual maturity in *D*. *virilis* occurs at about 5 days. To determine the dynamics of TE expression as flies aged, we analyzed mRNA-seq data from 0–2 hour old embryos laid by F1 mothers 12–16 days old, and 19–21 days old.

**Fig 2:**
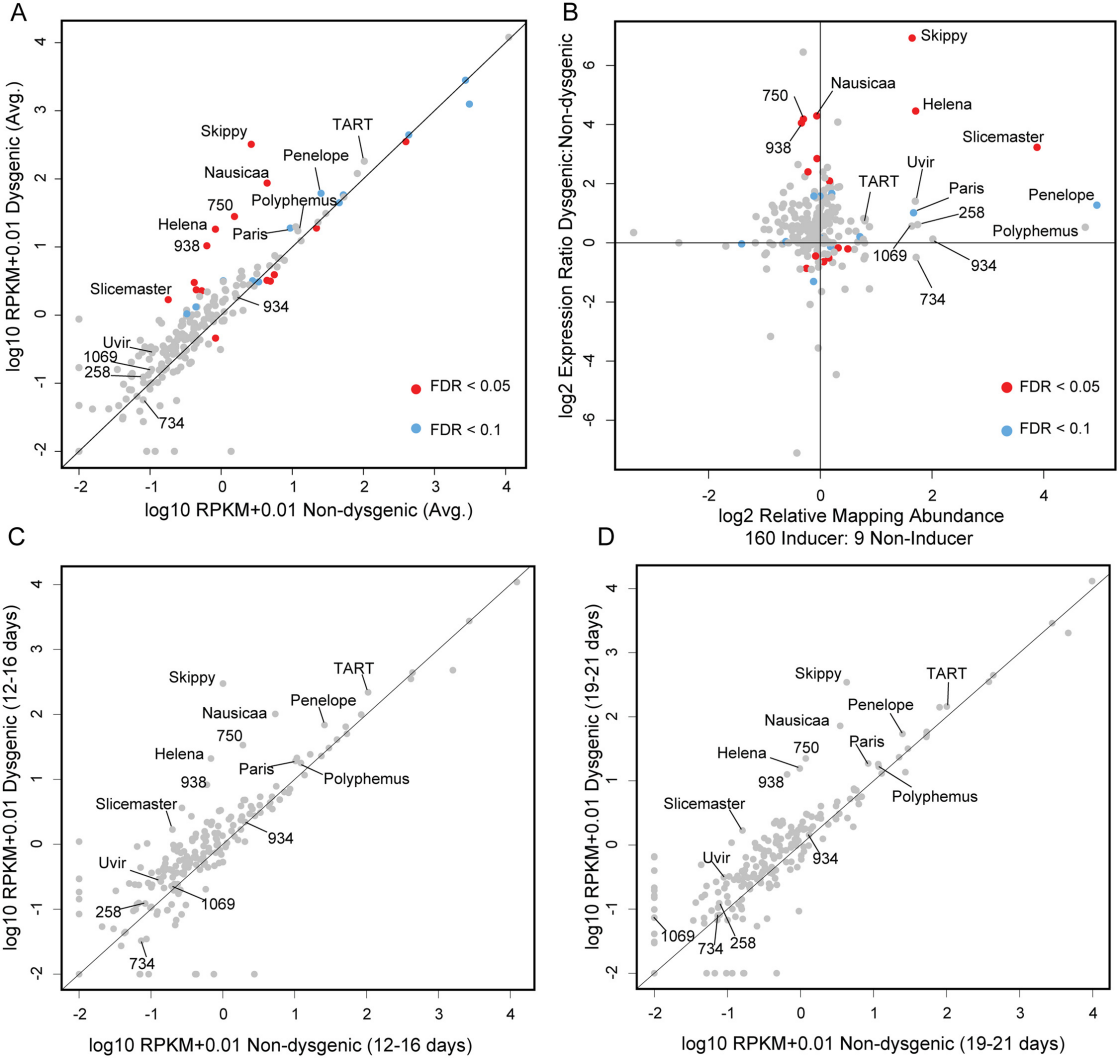
Increased TE expression in the dysgenic germline persists through adulthood. (A) RPKM+0.01 (log 10, average across both ages) for TEs, Dysgenic vs. Non-dysgenic germline. TEs that are in excess in 160 are more highly expressed, as well as many TEs that are not in excess. (B) Fold excess in expression (RPKM+0.01, log 2, average across both ages) vs. fold excess in abundance in strain 160. Nearly all TEs that are in excess in 160 show increased expression in the dysgenic germline (11/12). But multiple TEs that are equivalent in abundance between strains are also increased in expression. (C,D) Increased expression in the dysgenic germline is maintained as flies age. Note: Log scale obscures magnitude of difference for some TEs that demonstrate significant differences in expression identified due to low variation across replicates.

First, ignoring age effects, our mRNA-seq results indicated different modes of increased TE expression in the dysgenic germline (Fig 2A). Overall, we find 15 TEs that were differently expressed between dysgenic and non-dysgenic germlines (FDR<0.05). Of these, nine are significantly up in dysgenic and six are significantly up in reciprocal females, but the magnitude of increased expression in the dysgenic germline is significantly greater (Mann-Whitney U test comparing magnitudes of expression difference among differentially expressed TEs: U = 0, p<0.05). This is shown by the fact that all nine of the TEs with significantly increased expression in the dysgenic germline show more than a two-fold increase, but none of the six that are higher in expression in non-dysgenic progeny show this level of expression difference.

TEs with excess abundance in the inducer strain showed increased expression in the dysgenic germline. There were high magnitude differences for some elements (*Skippy* and *Helena*; Fig 2A) but for others, the observed differences in expression between dysgenic and non-dysgenic germlines were more modest (*Paris*, *Polyphemus* and *TART;* Fig 2A.) Importantly, while an assemblage of TEs are more highly expressed in the dysgenic germline, many TEs are expressed at equal levels in dysgenic and non-dysgenic crosses. This is also seen for TE expression in the *P-M* system [28].

Overall, there is not a general rule that *all* elements more highly expressed in dysgenesis are higher in copy number in the inducer strain (Fig 2B). Ten of eleven elements that are more abundant in the inducer strain were expressed at higher levels in dysgenic progeny. However, five of the nine elements with significantly higher expression in the dysgenic germline are in slightly higher copy number in strain 9 (Fig 2B). This finding is consistent with the observed co-mobilization that was originally identified through genetic approaches—TEs with similar copy number between strains can be co-mobilized by dysgenesis. For example, the *Ulysses* element is in similar copy number between strains, mobilizes in dysgenesis and shows about a two-fold increase in expression in the dysgenic germline, though this expression difference was not significant. In contrast, the *Telemac* element, at near-equal abundance between strains 160 and 9, was one of the five expressed at slightly higher levels in the non-dysgenic germline.

Accounting for age of F1 mothers, we found that the observed patterns of increased TE expression in the dysgenic germline were maintained through adulthood (Fig 2C and 2D). For example, the *Helena* element, which is more abundant in the inducer strain, shows an approximate 30-fold higher expression in embryos laid by 12–16 day old mothers. And in embryos laid by 19–21 year old mothers, *Helena* maintains an approximately 15-fold higher expression. This persisting level of increased TE expression in the dysgenic germline stands in contrast to the *P-M* system, where by 21 days, *P* element expression equalizes between dysgenic and non-dysgenic females [28].

### Maternal piRNA and siRNA deposition is an inconsistent predictor of TE expression in dysgenesis

To determine how maternal inheritance of piRNAs (defined as small RNAs, 23–30 nt, filtered against known non-piRNA classes) and also siRNAs (defined as small RNAs, 21 nt, filtered against known non-siRNA classes) might explain increased and persistent TE expression in the dysgenic germline we sequenced 18 to 30 nt RNAs from 0–2 hour old embryos laid by strain 9 and strain 160 mothers, and by F1 females from reciprocal crosses between the two strains. For F1 germline small RNAs, we collected embryos from the same pool of mothers used for mRNA-seq, but at intermediate maternal age (15–16 days old). This allowed us to determine whether the persistent differences in TE expression in the F1 germline of the dysgenic cross could be explained by a persistent defect in piRNA biogenesis.

**Fig 3:**
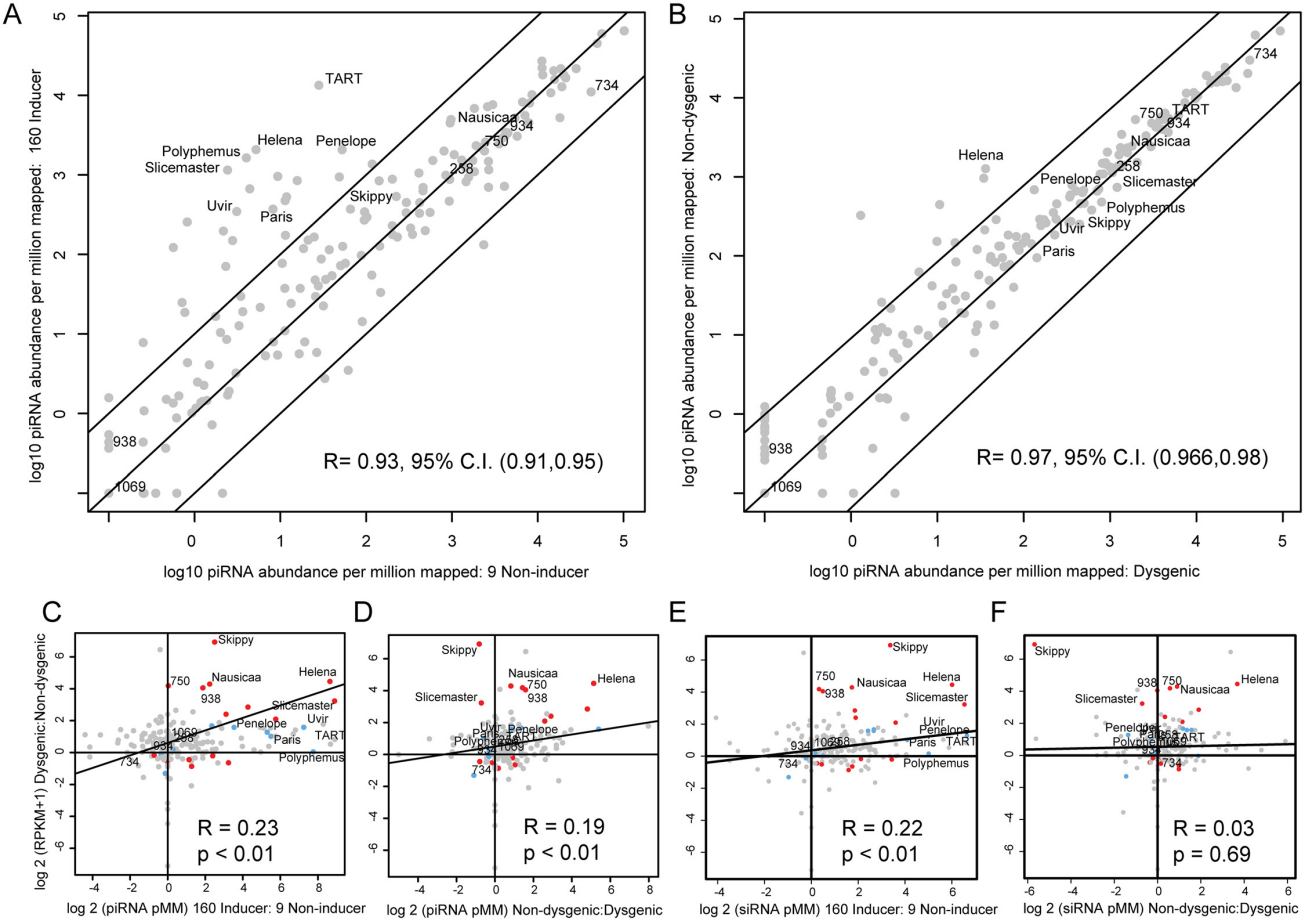
TE expression as a function of piRNA and siRNA abundance in parental strains and progeny. (A). Normalized (per 1 million mappers) piRNA abundance +0.1 (log 10) in the strain 9 germline vs. the strain 160 germline. A large number of TEs show increased piRNA expression in the strain 160 germline, especially TART and others enriched in abundance in strain 160. Diagonal lines indicate 10-fold levels of difference (B) Normalized (per 1 million mappers) piRNA abundance +0.1 (log 10) in the dysgenic germline vs. the non-dysgenic germline. piRNA abundances for many TEs with greater excess in strain 160 become similar in the dysgenic germline. A significant exception to this is the *Helena* element. Diagonal lines indicate 10-fold levels of difference. (C) TE piRNA excess in strain 160 vs. relative expression level in dysgenesis. TE piRNA asymmetry between 160 and 9 is not the sole determinant of increased expression in dysgenesis. Some elements, such as 750, are increased in expression in dysgenesis, despite similar piRNA abundances in 9 and 160. (D) TE piRNA excess in the non-dysgenic germline vs. relative TE expression level in dysgenesis. Elements such as *Skippy* and *Slicemaster* show equilibrated piRNA abundances, but excess expression in the dysgenic germline. (E) TE siRNA excess in strain 160 vs. relative TE expression level in dysgenesis. (F) TE siRNA excess in the non-dysgenic germline vs. relative TE expression level in dysgenesis.

A large number of TEs, including many with greater copy number in Strain 160, showed higher levels of maternally provisioned piRNA in strain 160 compared to strain 9 (Fig 3A). However, many TEs without large differences in copy number between strains also showed a more than 10-fold excess of maternally provisioned piRNA in the strain 160 genetic background (Fig 3A). Strikingly, despite the asymmetry in maternal provisioning observed in the strain 160 compared to the strain 9 background, piRNA differences are much less pronounced in the germlines of F1 individuals derived from the reciprocal 160 x 9 crosses (Fig 3B, non-overlapping 95% C.I.s for Pearson's correlation coefficient comparing to Fig 3A). This result is not consistent with global persistence of the maternally provisioned piRNA profile across generations. A significant exception to this is the *Helena* element, which maintains a higher level of piRNA in the non-dysgenic germline.

Fig 3C demonstrates the degree to which asymmetry in maternal provisioning predicts TE expression in reciprocal dysgenic and non-dysgenic progeny in the next generation. Many of the elements that are more abundant in strain 160 have greater piRNA abundance in the 160 female germline and also are expressed at higher levels in the germline of the dysgenic cross. This drives a significant positive correlation between the log 2 of the ratio of piRNA abundance between strains and relative TE expression levels. However, with Spearman's rho less than 0.2, this is not a strong relationship. Therefore, maternal provisioning of piRNA is only a modest predictor of TE expression in hybrid dysgenesis. For example, element 750 shows no difference in piRNA abundance between parental strains but is more highly expressed in the dysgenic germline. Additionally, a reduced level of TE piRNA abundance persisting in the dysgenic germline is positively associated with increased expression for many (Fig 3D, for example *Helena*), but not all elements. In contrast to *Helena*, *Skippy* and *Slicemaster* are both more highly expressed in the dysgenic germline even though both show *higher* levels of piRNA abundance in the dysgenic germline. Therefore, multiple mechanisms appear to explain increased TE expression that persists in the dysgenic germline.

We also examined the role that siRNAs had in predicting differences in TE expression between reciprocal progeny. Similar to the effect of piRNA provisioning, we found that differences in the maternal load of TE siRNAs were predictive of differential TE expression between dysgenic and non-dysgenic progeny (Fig 3E). Since differences in maternal piRNA and siRNA load are strongly correlated between the strains (S1 Fig), this is not surprising. Interestingly, in contrast to piRNA levels, zygotic siRNA levels were not predictive of differences in TE expression between reciprocal progeny (Fig 3F). Overall, maternal piRNA levels are predictive of F1 piRNA levels but maternal siRNA levels are not predictive of F1 siRNA levels (S1 Fig). Interestingly, maternal piRNA levels are in fact predictive of F1 siRNA levels (S1 Fig). These results suggest that while siRNA levels are poor predictors of TE expression differences, their biogenesis in F1 progeny may be coupled to piRNA abundance.

### Increased and persisting dysgenic TE expression is not associated with a collapse of global piRNA biogenesis

**Fig 4:**
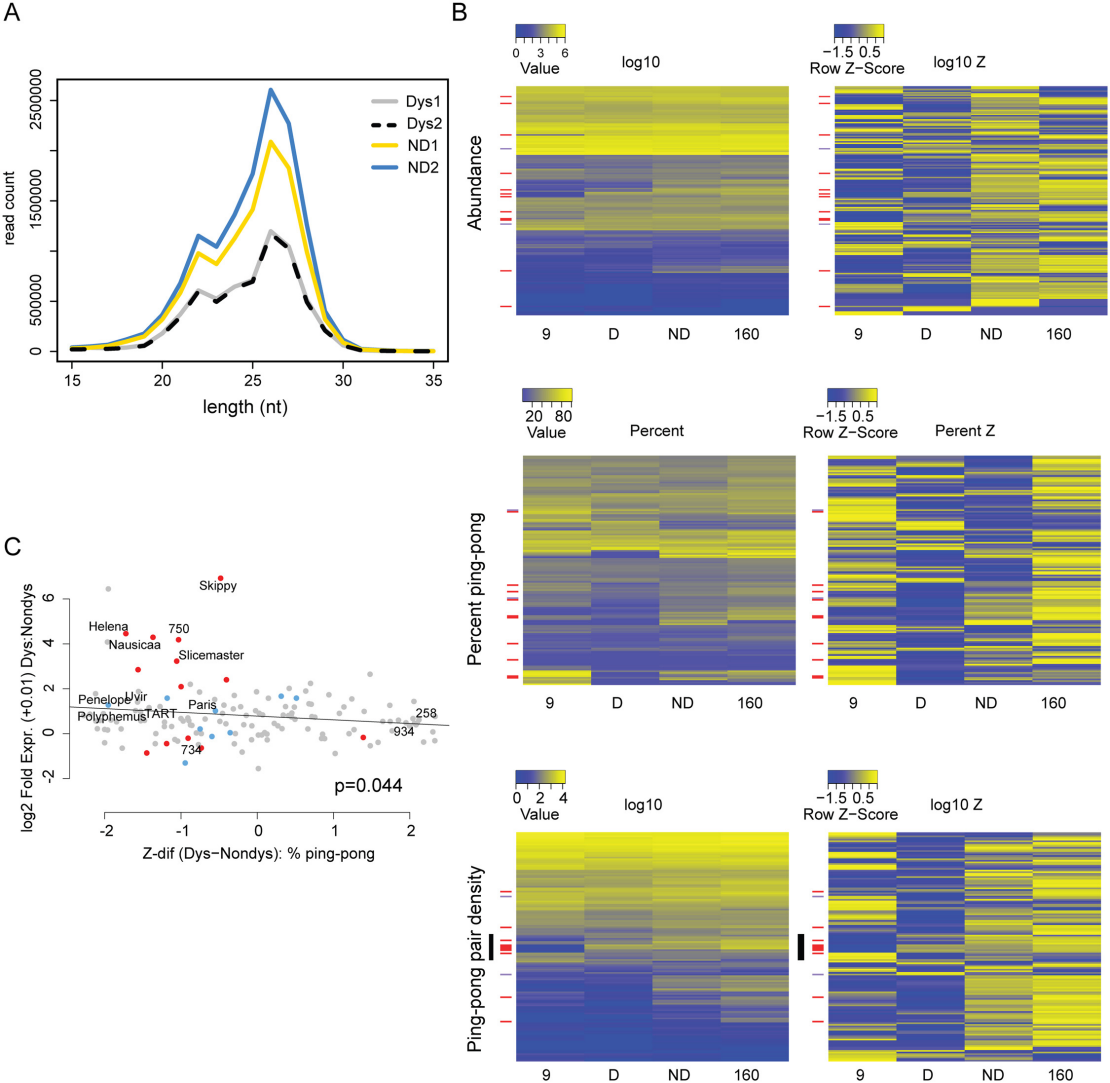
Signatures of piRNA biogenesis in the dysgenic germline show only modest defects. (A) Size distributions of small RNAs are similar between dysgenic and non-dysgenic germlines. Distribution of all small RNAs (not normalized) from four germline libraries (2 dysgenic, 2 non-dysgenic) filtered for tRNA, rRNA and snoRNA. (B) piRNA biogenesis signature heatmaps. TEs upregulated in the dysgenic germline (a difference of 5 RPKM or higher) are indicated with red bars. TEs upregulated in the non-dysgenic germline (a difference of 5 RPKM or higher) are indicated with purple. On the left are heatmaps for raw measures of abundance, the density of ping-pong pairs and percent ping-pong. On the right are heatmaps for the same metrics, but by row z-score. For raw measures, there are no globally discernible effects of dysgenesis on piRNA biogenesis. Row z-scores in dysgenesis do show lower values for abundance measures (abundance and ping-pong pair density), but not percent ping-pong (see text). (C) Fold excess in expression in dysgenesis vs. the difference in percent ping-pong Z-score between dysgenic and non-dysgenic germline. Of the top eight that are most differently expressed in dysgenesis, all have lower ping-pong z-scores in dysgenesis.

Raw abundance measures of piRNAs ignore critical aspects of their biogenesis and recent studies have demonstrated that globally reduced signatures of robust piRNA biogenesis likely contribute to the mobilization of diverse TEs [15,28]. In contrast to interspecific crosses that show near complete collapse of the 23–30 nt small RNA pool, we found no evidence that piRNA biogenesis is skewed away from the 23–30 nt expectation based on the size distribution of small RNA reads (Fig 4A). For each TE, we estimated the percent ping-pong [24] as well as the normalized density of ping-pong pairs in the dysgenic and non-dysgenic germline. When we compared metrics directly (first column of heatmaps, Fig 4B) we found little evidence that piRNA biogenesis is grossly perturbed in the dysgenic cross, though a more sensitive comparison using normalized Z-scores indicated a modest reduction in piRNA abundance and density of ping-pong pairs (Fig 4B). This is observed in the Z-score heat maps (Fig 4B, second column of heatmaps) for abundance and ping-pong pair density. Both showed an excess of negative Z-scores for dysgenesis (p<0.0001, Wilcoxon Signed-Rank Test). Importantly, both ping-pong abundance and ping-pong pair density are normalized, proportional measures of abundance that are likely influenced by increases in the abundance of non-TE, genic piRNAs in the same library (see below).

In contrast to the piRNA abundance measures, we found no significant evidence for global ping-pong biogenesis disruption as measured by percent ping-pong. Note, for example, that many row Z-scores for percent ping-pong showed weaker Z-scores for non-dysgenic compared to dysgenic piRNA (compare upper and lower portions of the percent ping-pong Z-score heatmap, Fig 4B). A Wilcoxon Signed-Rank test also found no significant difference (p = 0.06) in percent ping-pong Z-score between dysgenesis and non-dysgenesis piRNA. If there is any tendency for perturbed piRNA biogenesis, it is not uniform across elements.

Overall, there is a significant relationship between the difference in the normalized density of ping-pong pairs between parents and the difference between dysgenic and non-dysgenic germlines (S2 Fig), demonstrating a role for maternal provisioning in establishing piRNA biogenesis in the next generation. In light of this, we found that transposable elements more highly expressed in dysgenesis (red bars, Fig 4B) are in excess among elements with a reduced percent ping-pong signature. In particular, we found a significant correlation between difference in percent ping-pong Z-score and fold TE expression between dysgenic and non-dysgenic germlines (p = 0.044, Fig 4C). This trend is driven by the top eight elements that show higher expression in dysgenesis and all have lower percent ping-pong Z-scores in dysgenesis (Fig 4C). These results do not support a model of global disruption in piRNA biogenesis maintained in adult flies. Rather, they support a model in which the persistence of higher expression for some TEs is driven by idiosyncratic defects in the restoration of piRNA biogenesis in aged females that occur on a TE-by-TE basis. Strikingly, for several TEs, signatures of piRNA biogenesis appear largely restored, despite increased expression in the dysgenic germline. For TEs that are increased in expression in the dysgenic germline, there appear to be multiple causes (Table 1), including, but not limited to, reduction in ping-pong pairs, suggesting multiple modes of TE derepression in hybrid dysgenesis.

**Table 1:** Properties of TEs significantly more expressed in the dysgenic germline by at last 2-fold (FDR<0.1).

TE	160:9 Abund. (Log 2)	160:9 piRNA (Log 2)	Expression: D (RPKM)	Expression: ND (RPKM)	PP pair density: D (pMM)	PP pair density: ND. (pMM)	TYPE
*Helena*	1.71	8.64	18.25	0.83	0.31	53.04	I
*495*	0.21	2.35	1.05	0.33	37.79	119.30	I
*967*	0.17	5.75	2.28	0.53	0.21	6.72	I
*Penelope*	4.95	5.30	61.48	25.42	11.19	32.06	I
*Slicemaster*	3.88	8.89	1.69	0.18	12.63	13.89	II
*Skippy*	1.64	2.50	321.77	2.65	74.92	47.03	III
*Paris*	1.67	5.50	18.94	9.36	12.22	6.25	III
*Nausicaa*	-0.06	2.24	86.90	4.43	14.19	44.83	IV
*656*	-0.01	7.25	3.20	1.07	0.34	13.46	IV
*1012*	-0.06	4.27	3.01	0.42	0.27	52.99	IV
*190*	-0.12	3.53	1.32	0.44	16.66	45.73	IV
*620*	-0.22	3.11	2.35	0.45	0.00	9.93	IV
*938*	-0.34	1.87	10.42	0.63	0.00	0.00	V
*750*	-0.30	0.03	28.03	1.54	7.17	12.84	VI

160:9 Abundance, 160:9 total piRNA, Expression: D (Dysgenic, RPKM), Expression: ND (Non-dysgenic, RPKM), Ping-pong pair density: D (Dysgenic, per Million piRNAs mapped), Ping-pong pair density: ND (Non-Dysgenic, per Million piRNAs mapped). Type I: Higher in copy number in 160, higher in piRNA abundance in 160, F1 piRNA ping-pong pair density defined by maternal loading. Type II: Higher in copy number in 160, higher in piRNA abundance in 160, F1 piRNA ping-pong density equilibrated. Type III. Higher in copy number in 160, higher in piRNA abundance in 160, F1 piRNA ping-pong density higher in dysgenic. Type IV: Higher in copy number in 9, higher in piRNA abundance in 160, F1 piRNA ping-pong pair density defined by maternal loading. Type V: Higher in copy number in 9, higher in piRNA abundance in 160, no ping-pong pairs detected. Type VI: Higher in copy number in 9, equivalent piRNA abundance in 9 and 160, F1 piRNA ping-pong density higher in non-dysgenic.

To distinguish among the drivers of TE expression differences between the dysgenic and non-dysgenic germline, we used the *leaps* package in R to identify the single variable that was the best predictor within a multiple regression framework. We considered copy number difference between the two strains, differences in piRNA and siRNA abundance in parents and progeny, and differences in percent ping-pong. Differences in maternal piRNA abundance was selected as the single best predictor (*R-squared*: 0.054, *p =* 0.0005). Using an alternate approach to model selection using the AIC (stepAIC from the *MASS* package), the best fit model included only two variables: maternal siRNA abundance and piRNA abundance in the progeny (Multiple *R-squared*: 0.0674, *p =* 0.0005). Both of these variables are correlated with maternal piRNA abundance. Nonetheless, the selection of these two variables without including the maternal piRNA variable suggests that the influence of maternal piRNA abundance in the single variable model is jointly mediated by maternal siRNA pools and zygotic piRNA pools. It should be noted, however, the amount of variance explained by these models is low.

### Many genes are differentially expressed in dysgenic and non-dysgenic germlines

We found that 267 genes are significantly upregulated, and 300 significantly downregulated in the dysgenic compared to non-dysgenic germline (FDR = 0.05). We performed GO analysis using the GOrilla program that identifies enriched ontologies in sorted lists [50]. Sorting by FDR value, we identified several interesting terms for genes down-regulated in dysgenesis. These include: reproductive process (FDR = 0.00015), chromatin organization (FDR = 0.0015) and gene silencing (FDR = 0.037) (S1 Table). Since chromatin marks and gene silencing are important for TE control, down-regulation of these genes may play a role in increased TE expression in the dysgenic germline. However, a large number of GO terms were identified in the set of down-regulated genes and GO analysis can be difficult to interpret [51].

### Increased genic piRNA production in the dysgenic germline

Recent studies have demonstrated that in addition to TEs, genes may also be the target of piRNA silencing. Genic targeting by piRNAs can arise from neighboring TE insertions that drag flanking sequences into piRNA biogenesis and gene silencing in *cis* [52–57]. Previous work in the *D*. *virilis* system of dysgenesis identified a piRNA cluster overlapping the *center divider* (*cdi)* gene (Dvir\GJ14359) [35]. Global gene expression might be modulated if genic piRNA silencing were either attenuated or enhanced during dysgenesis. Therefore, we examined how the global landscape of genic piRNAs was influenced by the activation of diverse TEs in dysgenesis. For this analysis, we excluded genes that lacked hits to CDS regions. We also excluded genes that lacked orthologs in *D*. *melanogaster* to exclude TEs mis-annotated as genes.

**Fig 5:**
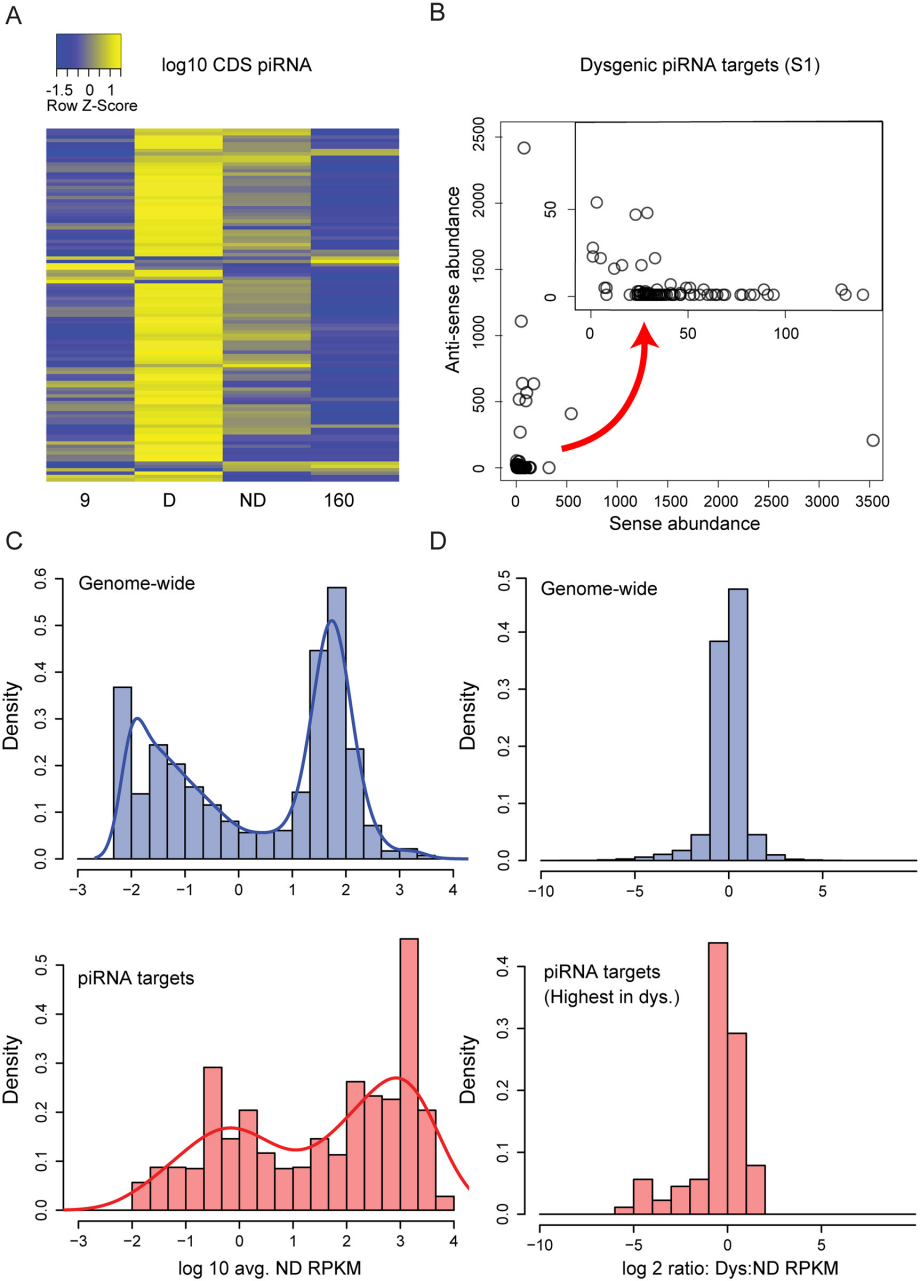
Genic piRNA targeting is increased in the dysgenic germline. (A) log10 Z-score heat map of genic (CDS) piRNA density for *D*. *melanogaster* orthologs (above a threshold of 5 piRNAs per CDS per 1 million mapped in at least one of four columns). Of these 105 genes, there is an excess of genic piRNAs in the dysgenic germline (89 genes with greatest genic targeting in dysgenesis, P<0.001) (B) Sense vs. Anti-sense abundance for piRNAs in genic piRNA class for one library (Sample 1). Some CDS regions are predominantly the source of anti-sense piRNAs, but the majority are biased as a source of sense strand piRNA (C) Distribution of expression levels (log 10 RPKM+0.01) for all genes in the genome and piRNA target genes (expression levels from non-dysgenic germline). Genic piRNA targets are derived from more highly expressed genes (p < 0.001). (D) Of 105 genes, the 89 that show excess genic piRNA in dysgenesis are also more lowly expressed in dysgenesis. Shown is the distribution of expression ratios (dysgenic:non-dysgenic) for all genes and genes that are increased as a source of genic piRNAs in dysgenesis (p < 0.001).

In the dysgenic germline we found significant enrichment of piRNAs derived from genes. We identified 105 genes that had at least 5 piRNA per million mapping to genic CDS regions in either the parental strains or reciprocal F1 progeny. For these 105 genes, there was a significant excess of piRNAs in the dysgenic germline (Fig 5A; p-value < 0.0001, Wilcoxon-signed rank test). Genic piRNA production, relative to parental strains, is also higher in non-dysgenic progeny, indicating that this may arise from crosses between strains with divergent piRNA profiles. For some genes, the genic piRNAs were predominantly anti-sense, but the majority of genes were associated with primarily sense strand piRNAs (Fig 5B). Comparing genic piRNA density across introns and exons, we found that piRNAs from these genes are enriched on exons (Paired T-test across genes contrasting intronic and exonic density: Library 1: p = 0.0046, Library 2: p = 0.0024). This suggests that genic piRNA processing may occur in the cytoplasm. We examined genic piRNAs for piRNA biogenesis signatures: first position U bias and 10th position A bias. Results indicate that the genic piRNAs are primary piRNAs (S2 Table). From the entire set of 105 genes, focusing on 80 genes that primarily produce sense piRNAs, we found a signature of primary piRNA biogenesis and a very weak signature of secondary piRNA biogenesis (Library 1: U first position: 0.33, background U: 0.21, A 10th position: 0.25, background A: 0.22; Library 2: U first position: 0.33, background U: 0.21, A 10th position: 0.24, background A: 0.23).

In addition, we looked for TE insertions within 2 kb upstream and downstream of these piRNA targeted genes. For the several genes that produced both sense and antisense piRNA, we often found evidence of either a TE in the reference genome or an indication of a nearby insertion in genomic mappings of one of the two strains. Not only did these genes have sense and antisense piRNA, but unique piRNA also mapped to intergenic regions around these genes, indicative of cluster spreading in *cis*. In contrast, genes with sense piRNA mapping only to exons usually showed no evidence of proximal TE insertions and piRNA did not map to intergenic regions. This supports the idea that these sense and exon-only piRNA are generated from processing of genic mRNA in the cytoplasm.

The 105 genes were also more highly expressed above the genome-wide background (Fig 5C, Wilcoxon rank sum test, p < 0.001). Among the genes with the greatest excess of piRNA abundance in the dysgenic germline, the primary piRNA biogenesis signature was strongest for the genes with expression level higher than 1000 RPKM (S2 Table). This indicates that these small RNAs are not simply degradation products of highly expressed genes. Importantly, the production of these genic sense piRNAs has an apparent effect on gene expression. Of the 105 genes, 89 were identified to show the highest piRNA abundance in the dysgenic germline. These 89 genes were more lowly expressed in the dysgenic germline compared to the non-dysgenic germline (Fig 5D, Wilcoxon rank sum test, p < 0.001). Among this set of 89 genes, there was a significant excess of genes more lowly expressed in dysgenesis compared to more highly expressed (p = 0.03, Sign Test). We attribute this to primary piRNA biogenesis from the sense strand since lower expression is observed for genes that are primarily targets of sense piRNA biogenesis (S3 Fig).

GO enrichment analysis using GOrilla indicated that these 105 genes are highly enriched for ribosomal proteins (FDR p-value = 1.9E-13, 19-fold enrichment; S3 Table). Seventeen of these eighteen ribosomal genes produce more piRNA in the dysgenic samples. These ribosomal piRNA targets are highly expressed, show a strong signature of primary biogenesis (S2 Table) and are not among the group that show differential mRNA expression between dysgenic and non-dysgenic samples. Notably, there is a gene with histone acetyltransferase activity, *nejire* (Dmel\nej), which has strong effects on TE expression upon knockdown in *D*. *melanogaster* [58] and is also orthologous to a piRNA target in our list (Dvir\GJ19060). *Nejire* produces more piRNA in the dysgenic samples than non-dysgenic, and has significantly lower mRNA levels in dysgenic versus non-dysgenic samples (p-adjusted value < 0.05).

### Pericentric regions rather than telomeres influence dysgenesis

While the induction of hybrid dysgenesis in *D*. *virilis* has been attributed to TEs enriched in the inducer strain 160, it has been proposed that differences in telomeric cluster activity in the inducer strain may also contribute [35]. Therefore, we employed a genetic approach to determine whether identified telomeric clusters were causal of dysgenesis or, perhaps, simply consequences of excess telomeric TART activity identified in the inducer strain. In the previous study [35], two telomeric clusters specific to strain 160 were identified, one residing at the tip of the second chromosome and another residing at the tip of the sixth chromosome. We therefore tested whether chromosomes carrying telomeres from the inducer strain 160 were highly inductive, when transmitted paternally, or highly protective, when present in the female germline of the dysgenic cross.

**Fig 6:**
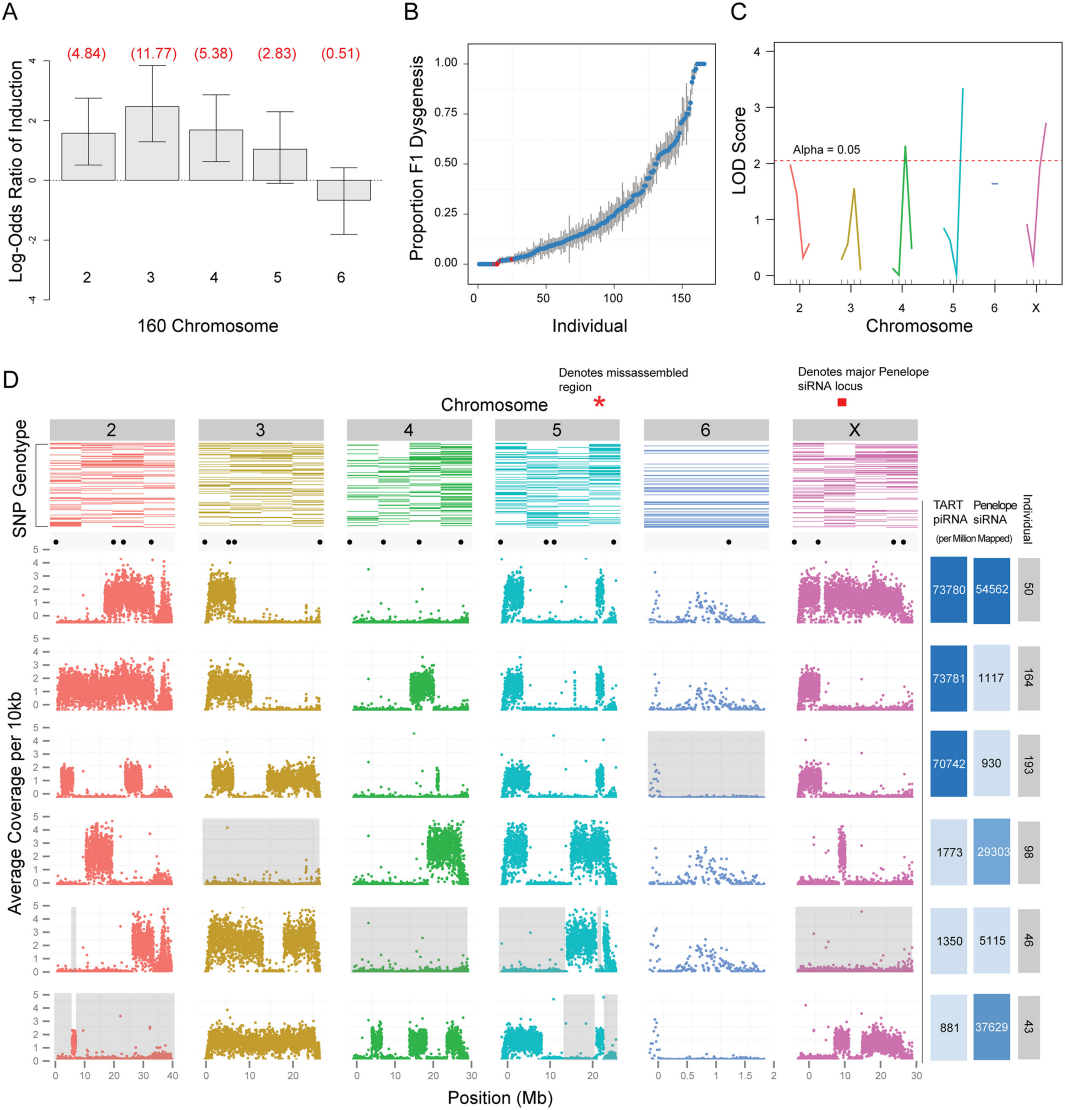
Genetic analysis of zygotic induction and maternal repression of gonadal atrophy. (A) Induction of sterility by 160 is broadly distributed across the genome, with the exception of chromosome 6 (the dot chromosome). Log odds ratios for probability of induction were estimated by crossing F1 males to strain 9, determining whether F2s had male gonadal atrophy and genotyping F2s to determine the chromosomes inherited from the father. Estimates were determined using a generalized linear model for logistic regression (binomial family with a logit link). Values in red are actual odds ratios. Whiskers are 95% confidence intervals. Chromosome 5 is significant at 0.1 level only. X chromosome is not scored because dysgenesis is scored in males and males do not inherit the X from their fathers (N = 92). (B) Scatterplot showing proportion of dysgenic testes (*y* axis) observed in the progeny of each F3 female individual (*x* axis). Red dots indicate F3 females that were selected for whole genome sequencing. (C) Single marker QTL analysis identified 3 putative QTLs: one flanking the centromeres of the 5^th^ and X chromosomes and one of the tested euchromatic regions of the 4^th^ chromosome. (D) Top row: Results from the genotyping assay. Colored rectangles represent the presence of strain 160 SNPs in individuals, ranked from top to bottom (most protective individuals on top). Scatterplots: sequencing results. Each dot represents the average number of base pairs that uniquely mapped to every 10kb of the 160 genome. Valleys indicate regions of strain 9 homozygosity. Black dots above scatterplots show the location of each SNP used for our genotyping assay. Grey background demonstrates that no region of the genome from 160 is necessary to protect against dysgenesis. Right-most columns: Number of piRNAs mapped to TART sequences, per million reads, for each F3 female individual. Color intensity is representative of TART piRNA abundance. Number of 21 nt endo-siRNAs mapped to *Penelope* sequences, per million 21 nt reads, for each F3 female individual. Color intensity is representative of *Penelope* endo-siRNA abundance. Red bar indicates position of one of several *Penelope* endo-siRNA loci on the X.

We found that induction of dysgenesis is distributed across all chromosomes, with the exception of the dot sixth chromosome (Fig 6A). Therefore, strain 160 telomeres of the second and sixth chromosomes do not contribute uniquely to induction of dysgenesis. Using QTL analysis (Fig 6B) with special attention to telomeric and pericentric regions (motivated by the fact that these genomic compartments often contain TE-rich piRNA clusters[23,26]), we identified three genomic regions for which strain 160 variants at these positions significantly protected against F1 sterility when present in the mother (*i*.*e*., dysgenesis; Fig 6C). The genomic region with the most significant effect corresponded to the pericentric region of chromosome 5. The pericentric region of the X chromosome also explained a significant proportion of variation in protective ability, followed by a euchromatic region in the proximal arm region of chromosome 4. We tested for interactions between these loci and saw no evidence for synergism (p-value for all interactions >0.2). Our previous work also found chromosome 5 to be the most protective, followed by the X and then chromosome 4 [49]. Together with the results from this study, there is strong genetic evidence that pericentric, cluster-derived piRNAs play a role in the protection against dysgenesis in *D*. *virilis*. By contrast, variation in telomeric repeat abundance between strains does not explain variation in protection against dysgenesis. Thus, telomeric piRNA clusters and amplified TART elements are likely a result of TE destabilization in the inducer strain rather than a driver.

### No single region or piRNA pool is protective against dysgenesis

To determine precise regions of the 160 genome critical for protection in a dysgenic cross, we performed whole genome sequencing of the six most protective F1 females for which DNA was available (Fig 6B and 6D). We found that among the most protective F1 females there was no single genomic region consistently derived from strain 160. In addition, across all six mothers, we identified at least one mother homozygous for strain 9 at each position of the genome (excluding unassembled regions). Therefore, no single genomic region appears critical for protection against dysgenesis.

To determine whether piRNA from any particular TE enriched in 160 was dispensable for protection, we sequenced small RNAs from the individual pairs of ovaries of the six most protective F1 females. We first reasoned that the only TEs that are candidate inducers of dysgenesis are those for which piRNA abundance is greater in strain 160 than in strain 9. Among the 221 repeats within the TE library (S1 Dataset), there are 141 that meet this qualification. We further reasoned that if any female with full repressive ability had piRNA abundances for a TE that were similar to strain 9, we could rule out that TE as a driver of dysgenesis. Of the remaining 141 candidate TEs, 88 TEs have at least one protective female that has normalized piRNA abundance derived from that TE less than or equal to strain 9. Thus, we were left with 53 candidate contributing repeats. Using these criteria, we were unable to eliminate any of the elements 3-fold enriched from strain 160. Therefore, we are unable to conclude that maternal loading of piRNA corresponding to a single TE can mediate protection against the induction of dysgenesis.

piRNA sequence data from the six most protective F1 females suggests a minimal role for the TART element piRNAs as mediators of maternal protection. This is because the vast majority of TART piRNAs in the protective mothers are derived from the tip of the X chromosome from 160 (Fig 6D) and three of the six females that are most protective against dysgenesis lack the X 160 telomere allele. Notably, the telomeric region of the X has special silencing properties in *D*. *melanogaster* that may explain the excess of TART piRNAs derived from this one genomic region [59–63], but in this system this region plays no role in protection against dysgenesis.

Using small RNA sequence data from the six most protective F1 females we characterized the genetic basis for *Penelope* endo-siRNA production that we had previously shown to be derived from the X-chromosome [49]. We confirmed that endo-siRNAs are not abundant when the X-chromosome from strain 160 is lacking (See individual 46; Fig 6D). These data also demonstrated that *Penelope* endo-siRNAs are contributed by several loci on the X chromosome (compare individual 98 to 43 and individual 43 to 50; Fig 6D). However, as one individual (46) lacking the X chromosome is protective against dysgenesis, we can confirm that *Penelope* endo-siRNAs play a minimal role in mediating maternal protection.

### Genic piRNAs influence gene expression across generations in multiple ways

Together, the above results support a model of hybrid dysgenesis driven by the mass action of multiple transposable elements. However, divergence in the TE repertoire between strains can also lead to divergence in the genic piRNA profile. This is because genic piRNAs can be produced when TE inserts flank genes [57].

**Fig 7:**
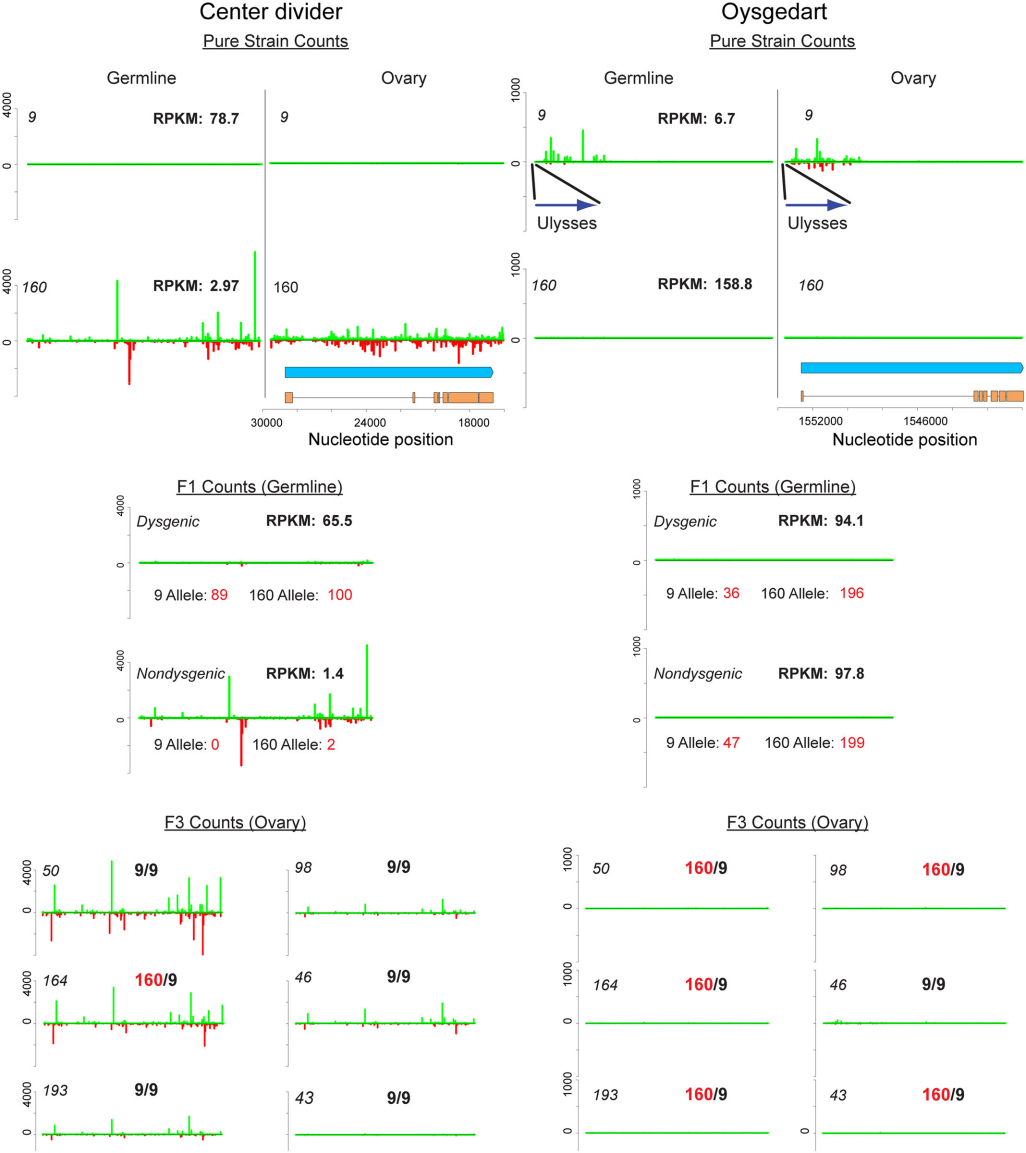
Germline and ovary genic cluster behavior across generations for *D*. *virilis* orthologs of *center divider* and *oysgedart* from *D*. *melanogaster*. piRNA mapping densities are indicated. mRNA-seq RPKM for germline (0–2 H embryo) is also indicated. Allelism was determined by counting mRNA-seq reads based on SNPs that distinguish strain 9 and 160. Strain 160 cluster identity is maintained for *cdi* in non-dysgenic progeny in which strain 160 is the mother. This is correlated with silencing of both alleles in the non-dysgenic germline. In contrast, the cluster is not maintained in the dysgenic germline and both alleles are expressed. Somatic expression is not affected. Germline cluster identity for *oysgedart* (which in the germline is predominantly sense) is lost in progeny. In this case, expression is even between reciprocal progeny, but germline expression is lower from the 9 allele in both directions of the cross. For cluster behavior in F3 backcrosses, heterozygosity or homozygosity of the respective allele is indicated. Notice how cluster identity is maintained for *cdi* to varying degrees in individuals homozygous for the 9 allele. In contrast, cluster activity is absent in all progeny for *oysgedart*.

Rozhkov *et al*. showed that strain 160 possesses a number of piRNA clusters absent in strain 9 [64]. The most well-characterized cluster is a telomeric cluster at the tip of chromosome 2 encompassing the gene *center divider (cdi)*. This dual strand piRNA cluster was found to be present in strain 160, but absent in strain 9. We observed the same pattern in our divergent laboratory stocks (Fig 7). For a second cluster identified by Rozhkov *et al*., near the telomere of the 6th chromosome in strain 160, but absent in strain 9, we found the opposite pattern in our strains. This cluster in our strain 160 had 318 unique mappers per million mapped reads, whereas our strain 9 had 3,836 unique mappers per million mapped. The most parsimonious explanation for this is that this cluster was originally present in both lines, but independently lost in our strain 160 and Rozhkov *et al*.*'s* strain 9. Thus, some piRNA clusters may be prone to losing their activity over time.

In addition to *cdi*, we identified the *oysgedart* gene (Dvir\GJ17620) in strain 9 that displayed a novel mode of genic piRNA targeting. A *Ulysses* element insertion upstream of this gene in strain 9 is specifically associated with genic silencing and shunting *oysgedart* into piRNA biogenesis (Fig 7). 5' genic piRNAs derived from *oysgedart* are dual-strand in strain 9 ovaries (which includes somatic and germline tissue) but biased towards the sense strand in the germline.

Maternal deposition of piRNAs derived from a specific locus can mediate piRNA biogenesis at that locus in the next generation [65,66]. For *cdi*, a recent study demonstrated that maternal deposition leads to maintenance of piRNA biogenesis from the 160 *cdi* allele. In progeny of strain 160 mothers and strain 9 fathers, maternally deposited piRNAs derived from the *cdi* cluster also activate piRNA biogenesis and methylation of H3K9 from the strain 9 *cdi* allele [67].

We found that two modes of genic targeting by piRNAs result in contrasting effects on gene expression across generations. The first mode was found at the *cdi* locus (Fig 7). Here, maternal deposition of *cdi* piRNAs mediates silencing in the germline, but not the soma. *cdi* gene expression is highly reduced in the germline of strain 160 and the germline silencing of both alleles of *cdi* is maintained when transmitted maternally and made heterozygous in combination with the wild-type strain 9 allele. In contrast, when the *cdi* cluster allele is transmitted paternally, piRNA cluster activity is reduced and germline expression levels are maintained near the level of strain 9, with similar contributions from each allele. Critically, this asymmetry in gene expression is not observed in the soma. Carcasses of females from reciprocal directions of the cross showed similar levels of expression from both alleles (Dysgenic Carcass: 17.1 RPKM [9 Allele: 7 counts; 160 Allele: 9 counts] vs. Non-dysgenic Carcass: 23.4 RPKM [9 Allele: 5 counts; 160 Allele: 12 counts]) (Fig 7).

The second mode was found at the *oysgedart* locus (Fig 7). The *oysgedart* allele in strain 9 shows a novel mode of genic targeting by piRNA. Similar to *cdi*, we identified sense and anti-sense piRNAs in the ovary. However, germline piRNAs derived from *oysgedart* are primarily sense derived. Strikingly, in neither direction of the cross is this form of sense piRNA biogenesis maintained in progeny. Instead, the wild-type allele from strain 160 seems to function in *trans* to limit this mode of silencing. Thus, in both directions of the cross, the expression of *oysgedart* is maintained at equal levels, but only at about 60% of wildtype since the *Ulysses* insertion allele from strain 9 is expressed at a lower level. While sense piRNA biogenesis is turned off in reciprocal directions of the cross, expression of the 9 allele is reduced in *cis*. In neither direction of the cross does there appear a maternal effect on somatic expression for *oysgedart*. Even though expression of the 9 allele is reduced in the germline, it remains on in the soma of both dysgenic (RPKM: 62.0, [9 Allele: 21 counts; 160 Allele: 21 counts]) and non-dysgenic (RPKM: 80.9, [9 Allele: 29 counts; 160 Allele: 25 counts]) F1 females (Fig 7). This demonstrates that the allelic *cis* effects on local silencing by the *Ulysses* insertion are germline, not soma, specific (Fisher's exact test for difference in allele effects between soma and germline. Dysgenic: p<0.0001, Nondysgenic: p<0.0001). Interestingly, in contrast to *cdi* which has shown to be enriched for H3K9 methylation in strain 160 ovaries, we find no evidence that *oysgedart* is so enriched in strain 9 ovaries (S4 Table) [67].

We then tested how this mode of piRNA biogenesis is maintained in further generations. We found that the *cdi* piRNA cluster can cause heritable activation of piRNA biogenesis from the strain 9 allele, a process equivalent to paramutation. In F3 flies generated by backcrossing hybrid non-dysgenic females to strain 9 males (maintaining maternal transmission of the cluster) cluster behavior was maintained even when *cdi* was homozygous for the strain 9 allele (Fig 7). In one case, cluster behavior was lost. Because the cross scheme was maintained over two generations, we are unable to determine the generation in which the cluster activity was lost, however, it is clear that *cdi* can paramutate in one generation but that this paramutation is not robust across multiple generations (though see [66]). In the same F3 flies, we did not observe restoration of cluster maintenance for *oysgedart*.

## Discussion

Due to their proliferative nature, differences in the TE landscape within populations and between species can accumulate rapidly. Since TEs are harmful, it has also been proposed that this divergence drives rapid evolution of the piRNA machinery itself [68–70]. Divergence at both levels is expected to greatly influence patterns of TE activity in crosses between individuals. Within a species, hybrid dysgenesis syndromes driven from a single element family reveal that maternally deposited piRNAs targeting the activating TE are critical for maintaining TE control and fertility. In contrast, crosses between species that differ with respect to both TE profile *and* the machinery of piRNA biogenesis show dramatic collapse of piRNA biogenesis and this can be attributed to divergence in the piRNA machinery rather than differences in the maternally deposited pool [15]. Previous studies, combined with results presented here, indicate that the *D*. *virilis* system represents a complex form of intraspecific hybrid dysgenesis involving more divergent TE profiles compared to other syndromes of hybrid dysgenesis. However, because it is an intraspecific cross, there is minimal divergence in the TE regulatory machinery. Therefore, the dysgenic syndrome in *D*. *virilis* can be considered an important model for understanding the dynamics of TE control at an intermediate stage in the divergence of TE profile within a single species.

Here we show that differences between genetically identical dysgenic and non-dysgenic individuals are manifested in multiple ways. When genomes from two strains of *D*. *virilis* are brought together, TEs that are more abundant in one genome become more highly expressed in the germline of the next generation. This difference in TE expression persists in the germline as flies age. Coincident with this, there is also a persistent increase in TE expression for several TEs that are evenly distributed between strains.

We identify multiple modes by which piRNAs modulate gene expression. First, many genes become off-targets for piRNA biogenesis, and their expression levels are reduced. The mechanism for this is unclear, but may be driven by the same mechanism that leads to idiosyncratic defects in piRNA biogenesis for some TEs. One possibility is that compromised piRNA function in the cytoplasm leads to a shift in the targets of primary piRNA biogenesis. A similar increase in sense genic piRNA abundance has been observed in *rhino* and *uap56* mutants and this has been attributed to compromised specificity in piRNA processing [71]. Therefore, the increased genic piRNA abundance in the dysgenic germline may be a readout of compromised piRNA biogenesis and loss of specificity in the cytoplasm. Secondly, divergence in the TE profile between strains leads to differences in the pool of maternally deposited genic piRNAs. Depending on the nature of genic piRNAs, these can modulate gene expression in diverse ways across generations. For the *cdi* gene, maternally deposited piRNAs from both strands mediate gene silencing of both alleles in non-dysgenic progeny. Alleles of *cdi* share properties with imprinted genes since expression depends on which parent the allele is inherited from. However, *cdi* differs from canonical imprinting in that a silenced *cdi* allele is capable of silencing the other allele in *trans* when inherited maternally, similar to paramutations observed in maize, mice and recently in *Drosophila melanogaster* [66,72–74]. Imprinted genes can have a significant downstream effect on patterns of gene expression [75]. Even in *Drosophila*, where there is no evidence for DNA methylation, there are significant, albeit poorly understood, parent-of-origin allelic effects on global gene expression [76,77]. Such transgenerational effects on gene expression may contribute to large numbers of genes being differentially expressed between the dysgenic and non-dysgenic germline. In contrast to *cdi*, a *Ulysses* insertion upstream of the *oysgedart* gene seems to mediate only deposition of sense piRNAs. In this case, the wild-type non-insertion allele appears to resist transgenerational silencing and expression is maintained equally between reciprocal hybrids.

Since we identify a large number of differences in TE and genic expression that are mediated by piRNAs in diverse ways, it is difficult to distinguish between causal factors and downstream effects. However, our genetic analyses clearly demonstrate that both induction of and protection against dysgenesis is distributed across the genome. Since multiple TE families are in excess copy number in the inducer strain, the weight of evidence favors a complex mode of hybrid dysgenesis driven jointly by the mass action of multiple elements. This is supported by the fact that the regions of the genome that are most protective against dysgenesis are located in the pericentric regions, which are known to be critical sources of piRNAs [23,26], further supporting a model in which pericentric regions play a unique role in TE control [78].

Representing an intermediate state of TE divergence between single TE family dysgenesis syndromes and interspecific crosses, several observations are worth noting from the *D*. *virilis* system. First, in crosses between *D*. *melanogaster* and *D*. *simulans*, piRNA biogenesis is globally defective and this is attributed to the accumulation of incompatibilities that arise from rapid divergence in the protein sequence of the piRNA biogenesis machinery. In the *D*. *virilis* system where there is only divergence in the TE profile between strains, we see no such global collapse of piRNA biogenesis. In this sense, this is more similar to what is observed in the *P-M* system of dysgenesis.

However, there are some key distinctions between the *P-M* system, driven by a single element, and the *D*. *virilis* system of dysgenesis. In the *P-M* system, as flies age, piRNAs targeting the active element are restored and *P* element silencing is regained. We see very little restoration of silencing for many TEs that are most likely to contribute to dysgenesis. For example, the *Helena* element remains much more highly expressed in the dysgenic germline and this is also associated with failure to restore the piRNA pool targeting *Helena*. Therefore, despite the fact that these tissues (0–2 hour old embryos) have escaped the ablation event of dysgenesis, idiosyncratic defects in TE silencing persist and these are passed on to the next generation. Furthermore, increased TE expression is observed for some TEs that appear to have a restored level of piRNA biogenesis. Thus, a further defect in dysgenesis is a lack of effective silencing by a restored pool of piRNA. This suggests multiple mechanisms contribute to chronic increased TE expression in the dysgenic germline.

A long-standing question is what underlies TE co-mobilization in dysgenic syndromes in *Drosophila*. For many years, the *D*. *virilis* system was considered unique in that it was associated with increased movement of elements equally abundant between strains, the *Ulysses* element being the best example. Recent work by Theurkauf and colleagues suggest that co-mobilization in fact may be a general property of dysgenic syndromes [28]. By what mechanism does the movement of one element activate the movement of others? The current working model is that DNA damage from a moving element activates the DNA damage response within the germline. This, in turn, drives Chk-2 mediated phosphorylation and degradation of Vasa. Vasa is critical for nuage assembly and germline piRNA biogenesis [79]. In the absence of proper Vasa and germline piRNA function, resident transposons become activated, leading to co-mobilization.

This is a strong candidate for the mechanism of co-mobilization in the *D*. *virilis* system. A burst of TE mobilization in the germline at any time during development may drive movement of TEs found in equal copy number between strains via the DNA damage response. However, it is difficult to explain the persisting and idiosyncratic pattern of increased expression for some TEs in the dysgenic germline that is observed in aged females that have escaped sterility. In the *P-M* system, Vasa degradation is relieved as the aging dysgenic flies recover partial fertility and *P* element silencing is restored. In *D*. *virilis*, full silencing is not restored, even in the germline that has escaped ablation. Coinciding with increased levels of expression that persist for some TEs, we also note that there is a persisting increase in piRNAs that target genes. This is similar to observations in flies defective in *rhino* and *uap56* [71]. We have shown that global defects in TE repression observed during hybrid dysgenesis share this feature. Strikingly, the genes that are piRNA targets are highly enriched for ribosomal proteins RNAs (FDR p-value: 1.9E-13, 19-fold enrichment; S3 Table). The mechanism by which increased genic piRNAs target ribosomal protein RNAs is unclear, however there are multiple lines of evidence that disrupted levels of ribosomal proteins can lead to increased levels of repeat expression [80]. For example, an early genome wide screen in *C*. *elegans* identified approximately 27 genes involved in transposon silencing, two of which were ribosomal proteins [81]. Disruption of the ribosome is known to trigger nucleolar stress and p53 activation [82]. Thus, nucleolar stress mediated by genic piRNAs may also contribute to persisting TE activation in the dysgenic germline.

## Materials and Methods

### Custom *D*. *virilis* TE library

Few annotated TEs are available for *D*. *virilis*. Therefore, we combined available annotated TE sequences with two computationally predicted libraries (generated with PILER [83] and REaS [84,85] ftp://ftp.flybase.net/genomes/aaa/transposable_elements/) to generate a manually curated library. Several annotations (*Uvir*, *Helena*, *TART*, *Telemac*) were improved by manual curation. Portions of the PILER library were also manually curated. Additional sequence from the *Helena* element was obtained by interrogating a *de novo* assembly of the strain 160 genome. Redundancy was removed from this combined library first by removing repeats with significant blastn hits between and within the PILER and annotated library, with priority to annotated and longer sequences. With this filtered set, further redundancy was removed by blasting this library with and between the REaS library.

### Genome Sequencing and TE measurement from strain 9 and 160

Genome sequencing was performed on both reactive strains 9 (non-inducer) and strain 160 (inducer). As *D*. *virilis* has very high satellite content (more than 40%) [86], we elected to used wandering third instar larvae for our DNA source as previously described [87]. These tissues include polytene tissues that are underreplicated in heterochromatin, thereby reducing the number of satellite reads and enriching for euchromatic TE insertions that are expected to be the most active. Wandering 3rd larvae were collected from strain 9 and 160, rinsed with 50% bleach, and DNA was extracted. 100 bp paired end sequencing was performed on an Illumina GAII with 400 to 500 bp fragments. TE abundance estimation was performed with single ends that were trimmed by Sickle (https://github.com/najoshi/sickle), mapped to the TE library with BWA-MEM [88] and normalized by read numbers mapping to the reference. Homogeneity within mapped reads was measured using piledriver (github.com/arq5x/piledriver) and averaging the frequency of the major allele across all nucleotides within the mapping for each TE.

### Estimation of zygotic effects of paternally inherited chromosomes

From the genome sequences of strain 9 and 160, restriction fragment length polymorphisms were identified that distinguish between the strains at two positions for each chromosome. F1 males were generated from a non-dysgenic cross and these males were crossed to strain 9. 96 F2 progeny were collected, (48 from each class, dysgenic or non-dysgenic) and genotyped for chromosomes inherited paternally with RFLPs. Log-odds ratios for the probability for being dysgenic with a given chromosome were estimated using a generalized linear model for logistic regression (binomial family with a logit link) in R. Some failed genotypes resulted in N = 92.

### mRNA seq: Dysgenic and Non-dysgenic germline, Strains 9 and 160 germline, dysgenic and non-dysgenic soma

#### Dysgenic vs. non-dysgenic germline

RNA for sequencing was collected from embryos laid by F1 mothers from the dysgenic and non-dysgenic directions of the cross. Ovaries were not selected because dysgenic ovaries are often atrophied. Therefore, the germline tissue represented in this experiment is derived from mothers that have escaped germline ablation. Paternal effects on embryos that might occur when dysgenic females are mated with sterile dysgenic brothers were minimized by equally mixing males from reciprocal directions of the cross and allocating them in mating cages between reciprocal F1 females. This also ensured improved egg laying from dysgenic females. Overall, we collected four pools of 0–2 hour old embryos, aggregated across several days, from large population cages containing many hundreds of adults grown up simultaneously from multiple bottles. Females were maintained as continuously laying with a constant supply of yeast and grape plates and eggs were collected after 0–2 hour durations and flash frozen in liquid nitrogen. RNA was pooled from collections of 12 to 16 day old mothers and 19 to 21 day old mothers. From each age sample, two RNA-seq libraries were generated for single-end, 50 bp sequencing, for a total of four libraries per condition (dysgenic or non-dysgenic).

#### 160 vs. 9 germline

The same strategy was employed for collection of 0–2 hour old eggs from strain 9 and 160. Here, pools were also aggregated over multiple collections from large cages of pure strain 9 and 160 from multiple grow up bottles. Pool were aggregated over 7 to 15 days (young) and 15 to 25 days (old) and split for two RNA seq libraries per pool with reciprocal barcodes. Results presented are average RPKM per library and allele counts were pooled across all libraries.

*Dysgenic and Non-Dysgenic Soma*: RNA for analysis from somatic gene expression was obtained from 3 dysgenic and 3 non-dysgenic crosses. From each cross, 10 females were collected and aged 3 to 9 days. RNA was collected from these pools of 10 females, with abdomens removed.

### Analysis of mRNA seq data

For estimates of gene expression level, RPKM was used. For statistical analysis, mapped count data was used. Reads were quality trimmed at the 3' end (up to 16 bp) and reads with 2 bp of quality less than 20 were excluded using the Galaxy server. TE RPKM estimates were obtained by directly mapping with BWA to the annotated TE library and normalizing with known TE length and the number of reads that mapped to the reference genome. mRNA RPKM estimates were obtained using the RNA-seq tool in CLC. Fold analysis was performed by calculating RPKM(+0.01) ratios. We used DEseq2 [89] on RNAseq read count data to test for differential expression for both TEs and genes between dysgenic and non-dysgenic germ lines. Analysis of TEs and genes was performed separately. The model employed tested for treatment effects, age effects, and age x treatment interaction effects. While the same model was fit for genes and TEs, these models were run independently to account for possible differences in the mean/variance relationship between these groups. Genes with fewer than 40 total reads mapped across all samples were removed from the analyses prior to calling differential expression in order to reduce the loss of power due to multiple testing. Statistical significance was assessed using FDR, focusing on treatment effect only. GO analysis was performed with GOrilla[50] using *D*. *melanogaster* orthologs genes sorted by FDR value for the test of treatment effect.

Additional germline mRNA seq was performed using the same protocol for pure strain 9 and strain 160 (RPKMs averaged for cluster analysis across 2 libraries each for 7–15 day old females and 15–25 day old females). Allele counts were determined by direct counting within the RNA-seq mappings (summed across all library mappings) for a SNP known to distinguish the two strains within the transcripts for *cdi* and *oysgedart*. Somatic mRNA seq analysis was likewise performed (3 libraries per condition, each library from a 10 pooled female carcasses, RPKMs averaged across libraries and allele counts estimated as before).

### Small RNA sequencing

All small RNA was size selected from 15% acrylamide, cut between an 18 bp oligo and the 30nt rRNA. Small RNA sequencing for dysgenic and non-dysgenic germline material was performed on embryos laid by the same mothers as for RNA seq, but at 15–16 days old, according to [90]. Small RNAs from strain 9 and strain 160 pooled ovaries were sequenced according to [91] with the oxidation reaction. Small RNAs from ovaries from individual F3 females was performed by Fasteris.

### Small RNA Analysis

Reads were trimmed by removing adapters and filtered by size as piRNA (23-30nt) in CLC Genomics Workbench 7.0. Reads were then filtered by mapping to tRNA, ncRNA, miscellaneous RNA, and miRNA (including pre-miRNA) libraries from the *D*. *virilis* reference genome. The filtered 23–30 nt small RNA reads were mapped to our curated TE library with BWA.aln [92], using the default parameters. Reads were normalized by non-unique mappers to the *D*. *virilis* reference genome using BWA.aln defaults. Calculations for ping-pong percent [24] and density of piRNA pairs were done with the R package viRome (http://www.ark-genomics.org/bioinformatics/virome), with some modifications. For genic small RNA analysis, reads were mapped uniquely with BWA.aln to the *D*. *virilis* reference genome, using default parameters. Reads were normalized by non-unique mappers to the genome. BEDTools intersect [93] was utilized to count piRNA hits on genes and CDS sequences. Fastq reads specific to genic piRNA hits were extracted using Enve-omics (https://github.com/lmrodriguezr/enveomics) and FASTX-Toolkit (hannonlab.cshl.edu/fastx_toolkit/index.html) was used to process and analyze nucleotide bias for genic piRNA subsets. We did not perform DEseq2 analysis because estimation of the dispersion parameter with DEseq2 is unlikely to be robust with about 200 TEs, compared to standard mRNA-seq analysis that estimates the dispersion parameter using thousands of genes [89]. Percent ping-pong was defined as the percent of 23 to 30 nt mapping reads that had a corresponding read on the opposite strand with a 10 bp 5'-5' overlap. We also measured piRNA biogenesis by determining ping-pong pair density. This measure was obtained by counting all non-redundant ping-pong pairs (counting each read only once) per kb.

### Genetic analysis of genomic regions from strain 160 that maternally protect against dysgenesis

To identify regions of the 160 genome that protect against dysgenesis when present in females, an F3 mapping/QTL experiment was performed. F3 females were generated by crossing 160 females to strain 9 males (a non-dysgenic cross), followed by two rounds of backcrossing to strain 9 males. This resulted in the production of F3 mothers for which strain 160 was the great-grandmother. All but the final cross was performed *en masse*. Dysgenic crosses were performed with >160 single 4 to 5 day old tester F3 females mated with three 4 to 5 day old strain 160 males. Adults were transferred to new vials daily and dysgenesis was estimated by counting the number of dysgenic testes in progeny over all testes counted (2 per male) across three broods. Females were then collected, ovaries removed (for small RNA sequencing by Fasteris, top protectors only) and carcasses retained for genomic DNA extraction. Genotyping was performed using the TaqMan Open Array platform on all females, with the exception of the top six females that had the strongest ability to protect against dysgenesis. SNPs distinguishing 160 and 9 chromosomes were chosen in pairs for redundancy, one pair at each telomere and pericentric region, as well as one or two euchromatic SNPs. Care was taken to avoid repeat sequences by screening with Blast against the reference and also using RepeatMasker. F3 Females were then genotyped for 160/9 heterozygosity, alongside pure strain 9 and 160 controls, by National Jewish Health. Single marker regression was carried out with RQTL after dropping individuals with missing genotype data from the analysis. 5000 permutations were done to find the significance threshold at alpha = 0.05. For the top 6 protectors, whole genome sequencing was performed (100 bp, paired-end) using the Nextera library prep protocol.

### Genotyping by whole genome sequencing

Reference genome scaffolds from *D*. *virilis* were concatenated according to their supported positions and orientations on known Muller elements, with a large scaffold arbitrarily generated by concatenating scaffolds from unknown positions. Using this new "assembly" we mapped all strain 9 and strain 160 reads and generated two consensus genomes. A pseudo-diploid heterozygous genome was then assembled by placing these scaffolds into one file.

Paired-end reads from the top six protectors were mapped to the hybrid genome, using BWA’s default parameters, with the goal of inferring spans of heterozygosity for strain 160 by identifying reads that map uniquely, under high stringency, to the strain 160 haploid reference. The mapping output was piped into SAMtools for filtering by quality score (-q 42) and post-alignment processing (SAM to BAM conversion and indexing). A relatively high cutoff quality score was used in order to remove reads that could have mapped promiscuously. We were able to remove all reads that mapped to more than one locus/allele leaving us with reads that are specific to either strain 9 or 160. Spans of heterozygosity for strain 160 were visualized with a sliding window for read density along the 160 chromosomes within the psuedo-diploid reference genome.

### Chromatin immunopreciptiation sequencing

Chromatin isolation and immunoprecipitation was performed using 200 pairs of ovaries from strain 9 and strain 160 according to the protocol described in Du and Elgin [52]. Immunoprecipitation (IP) was performed using anti-H3K9me2 (Abcam 1220) at 1:100. Input and IP DNA was used for SR50-bp Illumina sequencing. H3K9-me2 enrichment was estimated using the IP:Input ratio of reads (with duplicates removed) uniquely mapping to the *cdi* or *oysgedart* locus (normalized to the total number of reads mapping in the library).

## Supporting Information

S1 Dataset Repeat library used in the analysis. (TXT)

S2 Dataset Properties of the focal repeats analyzed. (XLSX)

S1 Fig Relationships between maternal and zygotic abundance for piRNA and siRNA pools. Red indicates TEs with significant differences in expression at FDR<0.05. Blue indicates TEs with significant differences in expression at FDR<0.1. A) Log 2 of piRNA abundance ratio (160:9, per million mapped) vs. Log 2 of siRNA abundance ratio (160:9, per million mapped). B) Log 2 of piRNA abundance ratio (160:9, per million mapped) vs. Log 2 of piRNA abundance ratio (non-dysgenic:dysgenic, per million mapped). C) Log 2 of siRNA abundance ratio (160:9, per million mapped) vs. Log 2 of siRNA abundance ratio (non-dysgenic:dysgenic, per million mapped). D) Log 2 of piRNA abundance ratio (160:9, per million mapped) vs. Log 2 of siRNA abundance ratio (non-dysgenic:dysgenic, per million mapped). E) Log 2 of piRNA abundance ratio (non-dysgenic:dysgenic, per million mapped) vs. Log 2 of siRNA abundance ratio (non-dysgenic:dysgenic, per million mapped).
(TIF)

S2 Fig Differences in TE ping-pong pair density are influenced by maternal differences in ping-pong pair density. The difference in ping-pong pair density (per million mapped) between 160 and 9 (160 minus 9) vs. the difference in ping-pong pair density (per million mapped) between Non-dysgenic and Dysgenic (Non-dysgenic minus dysgenic). Larger differences in ping-pong pair density correspond to larger differences between dysgenic and non-dysgenic germline. However, many TEs differentially expressed show minimal differences in ping-pong pair density, either in parents or offspring. Red indicates TEs with significant differences in expression at FDR<0.05. Blue indicates TEs with significant differences in expression at FDR<0.1.
(TIF)

S3 Fig Average expression (RPKM) ratio for genes from 0–2 hour old embryos laid by dysgenic and non-dysgenic females. A) Genome wide expression ratio distribution (Dysgenic:Non-Dysgenic) and B) Distribution of expression ratios (Dysgenic:Non-Dysgenic) for genes producing sense piRNAs, with highest piRNA abundance in the dysgenic treatment.
(EPS)

S1 Table GO analysis for mRNA seq, comparing dysgenic and non-dysgenic germline, using GOrilla. (XLSX)

S2 Table piRNA biogenesis signatures for genic piRNAs. (XLSX)

S3 Table GO analysis for genes enriched for genic piRNAs, using GOrilla. (XLSX)

S4 Table H3K9-me2 ChIP-seq results for ovaries from strain 160 and 9. As in previous analysis, the *cdi* locus shows enrichment. In contrast, *oysgedart* does not.
(XLSX)
