# Supplementary figures and images for: piRNAs Are Associated with Diverse Transgenerational Effects on Gene and Transposon Expression in a Hybrid Dysgenic Syndrome of D. virilis
Source: PLoS Genet. 2015 Aug 4;11(8):e1005332. doi: 10.1371/journal.pgen.1005332 (PMC4524669; doi:10.1371/journal.pgen.1005332)

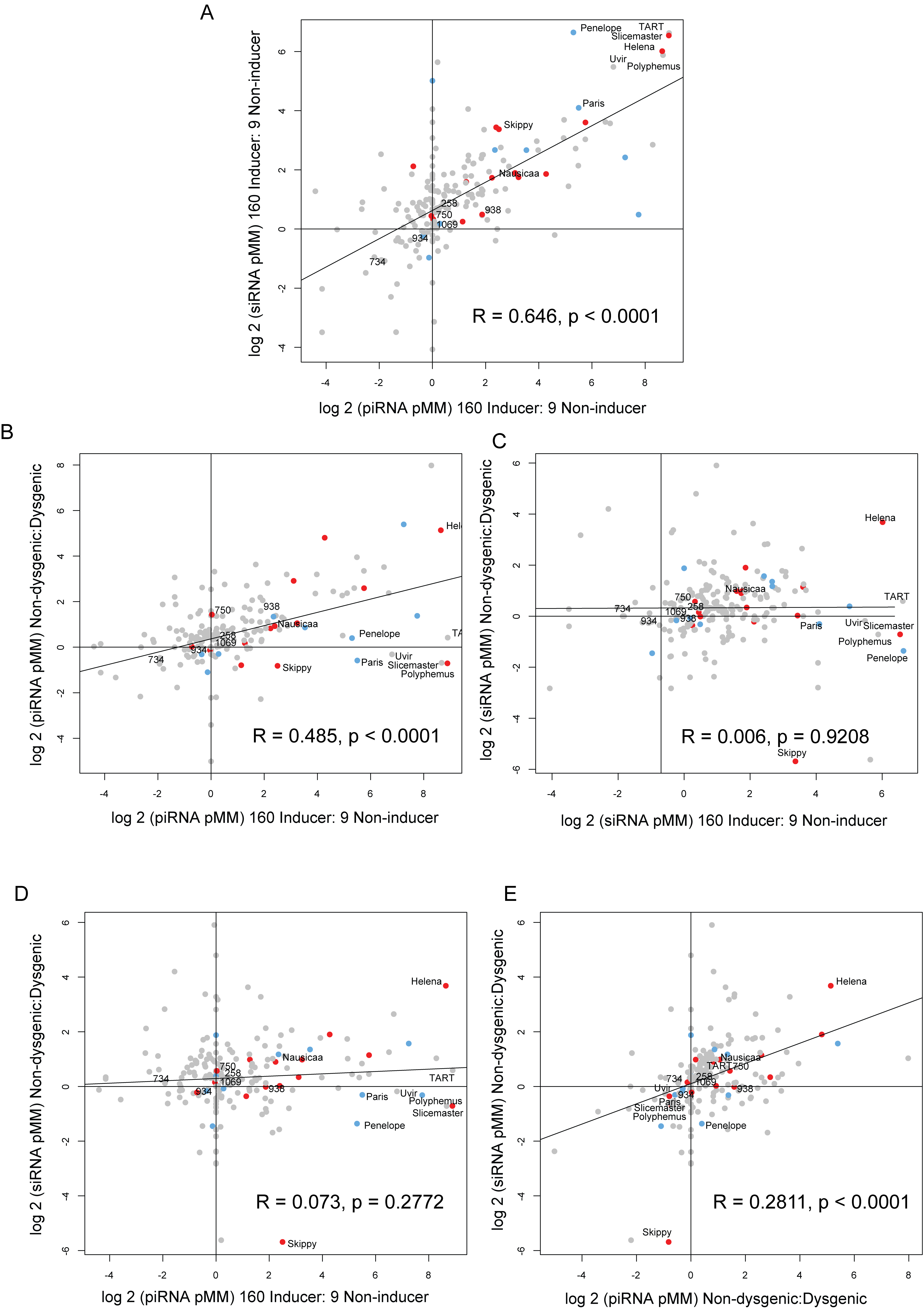

Supplement: S1 Fig — Red indicates TEs with significant differences in expression at FDR<0.05. Blue indicates TEs with significant differences in expression at FDR<0.1. A) Log 2 of piRNA abundance ratio (160:9, per million mapped) vs. Log 2 of siRNA abundance ratio (160:9, per million mapped). B) Log 2 of piRNA abundance ratio (160:9, per million mapped) vs. Log 2 of piRNA abundance ratio (non-dysgenic:dysgenic, per million mapped). C) Log 2 of siRNA abundance ratio (160:9, per million mapped) vs. Log 2 of siRNA abundance ratio (non-dysgenic:dysgenic, per million mapped). D) Log 2 of piRNA abundance ratio (160:9, per million mapped) vs. Log 2 of siRNA abundance ratio (non-dysgenic:dysgenic, per million mapped). E) Log 2 of piRNA abundance ratio (non-dysgenic:dysgenic, per million mapped) vs. Log 2 of siRNA abundance ratio (non-dysgenic:dysgenic, per million mapped). (TIF) [file pgen.1005332.s003.tif]

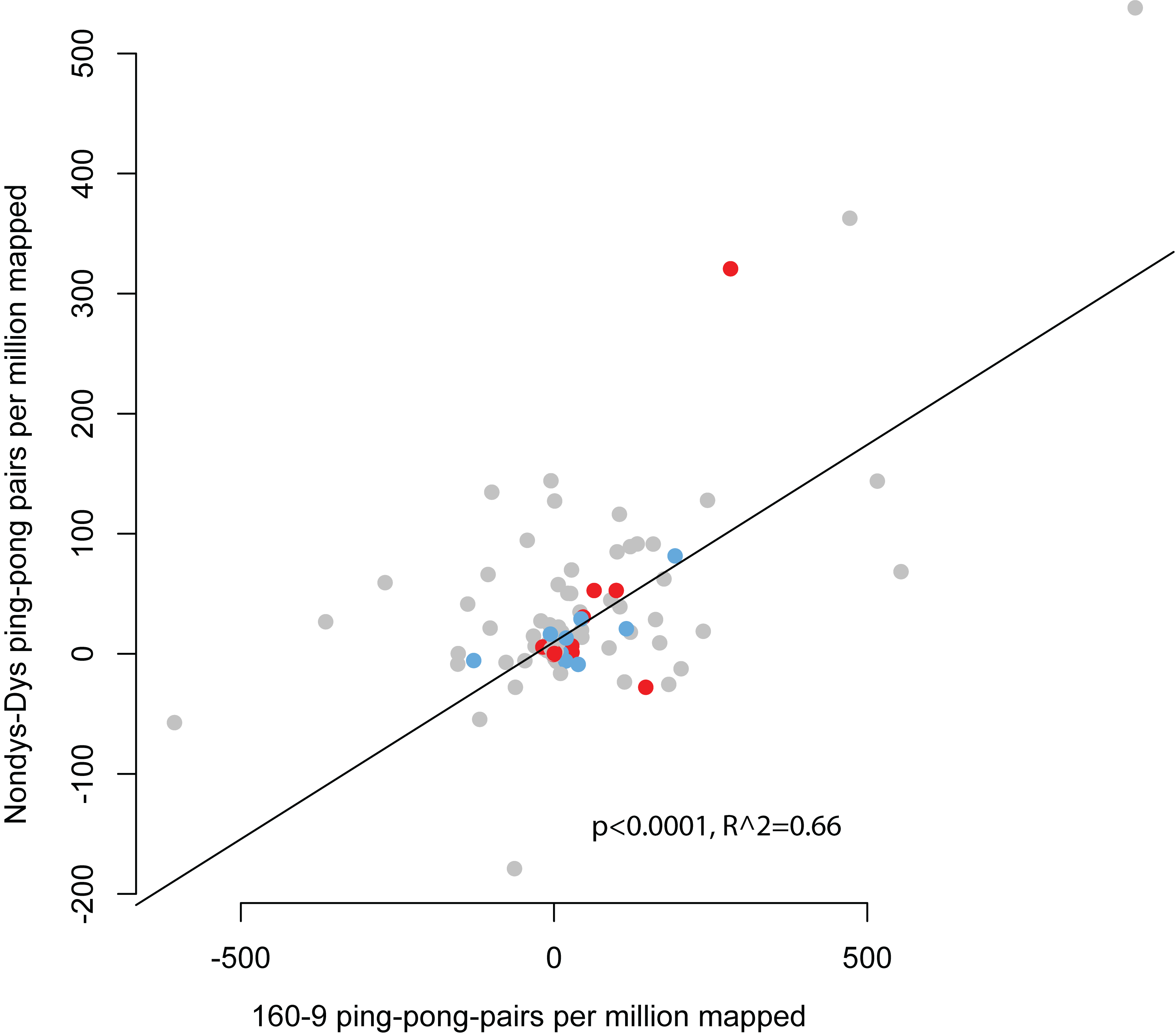

Supplement: S2 Fig — The difference in ping-pong pair density (per million mapped) between 160 and 9 (160 minus 9) vs. the difference in ping-pong pair density (per million mapped) between Non-dysgenic and Dysgenic (Non-dysgenic minus dysgenic). Larger differences in ping-pong pair density correspond to larger differences between dysgenic and non-dysgenic germline. However, many TEs differentially expressed show minimal differences in ping-pong pair density, either in parents or offspring. Red indicates TEs with significant differences in expression at FDR<0.05. Blue indicates TEs with significant differences in expression at FDR<0.1. (TIF) [file pgen.1005332.s004.tif]
